# HGF-Induced PKCζ Activation Increases Functional CXCR4 Expression in Human Breast Cancer Cells

**DOI:** 10.1371/journal.pone.0029124

**Published:** 2012-01-05

**Authors:** Songyin Huang, Nengyong Ouyang, Ling Lin, Lili Chen, Wei Wu, Fengxi Su, Yandan Yao, Herui Yao

**Affiliations:** 1 Department of Laboratory, Sun Yat-Sen Memorial Hospital, Sun Yat-Sen University, Guangzhou, China; 2 Department of Gynaecology and Obstetrics, Sun Yat-Sen Memorial Hospital, Sun Yat-Sen University, Guangzhou, China; 3 Breast Tumor Center, Sun Yat-Sen Memorial Hospital, Sun Yat-Sen University, Guangzhou, China; 4 Department of Rheumatology, The First Affiliated Hospital, Shantou University Medical College, Shantou City, Guangdong, China; 5 Department of Oncology, Sun Yat-Sen Memorial Hospital, Sun Yat-Sen University, Guangzhou, China; Vanderbilt University Medical Center, United States of America

## Abstract

The chemokine receptor CXCR4 and its ligand CXCL12 have been shown to mediate the metastasis of many malignant tumors including breast carcinoma. Interaction between hepatocyte growth factor (HGF) and the Met receptor tyrosine kinase mediates development and progression of cancers. HGF is able to induce CXCR4 expression and contributes to tumor cell invasiveness in breast carcinoma. However, the mechanism of the CXCR4 expression modulated by c-Met-HGF axis to enhance the metastatic behavior of breast cancer cells is still unclear. In this study, we found that HGF induced functional CXCR4 receptor expression in breast cancer cells. The effect of HGF was specifically mediated by PKCζ activity. After transfection with PKCζ-siRNA, the phosphorylation of PKCζ and CXCR4 was abrogated in breast cancer cells. Interference with the activation of Rac1, a downstream target of HGF, prevented the HGF-induced increase in PKCζ activity and CXCR4 levels. The HGF-induced, LY294002-sensitive translocation of PKCζ from cytosol to plasma membrane indicated that HGF was capable of activating PKCζ, probably via phosphoinositide (PI) 3-kinases. HGF treatment also increased MT1-MMP secretion. Inhibition of PKCζ, Rac-1 and phosphatidylinositol 3-kinase may attenuate MT1-MMP expression in cells exposed to HGF. Functional manifestation of the effects of HGF revealed an increased ability for migration, chemotaxis and metastasis in MDA-MB-436 cells *in vitro* and *in vivo*. Our findings thus provided evidence that the process of HGF-induced functional CXCR4 expression may involve PI 3-kinase and atypical PKCζ. Moreover, HGF may promote the invasiveness and metastasis of breast tumor xenografts in BALB/c-nu mice via the PKCζ-mediated pathway, while suppression of PKCζ by RNA interference may abrogate cancer cell spreading.

## Introduction

Identification of novel therapeutic targets is critical for the treatment of malignant tumors, breast cancers in particular [1], [2]. A quite exciting one of such targets is the chemokine (C-X-C motif) receptor 4 (CXCR4) expressed in various types of tumors [3]. In breast cancer cells, CXCR4 is a major chemokine receptor with possible but undefined roles in metastatic cell diffusion and homing to secondary sites [4]. Most models of CXCR4 functions in tumor focus on its potential role as a mediator of motility, invasiveness and metastatic behaviors [5], [6]. Blocking CXCR4 may attenuate breast cancer metastasis to regional lymph nodes and the lungs [7], and activation of CXCR4 is believed to be primarily ligand-dependent, as is supported by the antitumor efficacy of AMD 3100, an antagonist of chemokine (C-X-C motif) ligand 12 (CXCL12) binding [8]. CXCR4 is phosphorylated in response to ligand binding in a G protein-coupled receptor kinase 2-dependent fashion [9]. Phosphorylation of CXCR4 can also occur in response to the activation of other receptors and can involve additional kinases, such as protein kinase C ζ (PKCζ) [10], [11] or tyrosine kinases [12]. Regulation of phosphorylation and internalization has a significant effect on CXCR4-mediated cell responses [10]. Thus, understanding the mechanisms and molecular pathways that affect CXCR4 expression and cellular signaling might have important implications for breast cancer cell metastasis.

Hepatocyte growth factor/scatter factor (HGF/SF) is an important fibroblast-secreted protein that mediates development and progression of cancers [13]. HGF has been demonstrated to transduce its biological activities through the Met receptor tyrosine kinase by activating a number of intracellular pathways which in turn transmit the HGF signal to cytosol and to nucleus [14], [15], [16]. The HGF/Met couple controls the expression of a panel of genes that are important for these processes, including HIF-1a (the inducible subunit of HIF-1 transcription factor), members of the plasminogen activation system and the C-X-C motif receptor 4 (CXCR4) [17], [18]. The receptor tyrosine kinases Met and CXCR4 are associated with the morphogenesis and functional differentiation of normal mammary gland epithelium and play an important role in malignant transformation [19]. Previously, both CXCR4 and c-Met were shown to be upregulated by hypoxia in glioma cells and breast cancer cells [17], [18], [19], [20]. HGF up-regulates CXCR4 expression via NF-kappaB and contributes to cell invasion. Knock-down of NF-kappaB expression inhibited the induction of CXCR4 expression in response to HGF [21], [22]. In the cases of liver injury, HGF can promote the expression of CXCR4 in human CD34 stem cells, and subsequently, CD34 stem cells migrate to the injury site under the directional guidance of chemokine stromal cell-derived factor-1 (SDF-1, also named CXCL12). The protein kinase C family (PKC) has been shown to be involved in the expression and function of CXCR4, and is also linked to HGF-induced intracellular signal transduction in tumor cells. It is therefore vitally important to understand whether PKC pathway is associated with the expression and function of CXCR4 induced by HGF [13], [23].

A specific isoform of the PKC family, the atypical PKCζ, is an important class of secondary messengers that mediates a number of cellular responses to exogenous stimuli and stress agents. Unlike conventional (α,β,γ) and novel (δ, ε, η, θ) PKC isoenzymes, atypical PKCs(aPKCs) include PKCζ and PKCλ/ι which are not activated by calcium and diacylglycerol [24]. The atypical PKCζ is a key regulator of CXCL12/CXCR4-activated signaling in human hematopoietic progenitors. Petit I et al have demonstrated that ectopic PKCζ expression increases SDF-1 induced motility, whereas inhibition of PKCζ activity impairs survival, proliferation, adhesion and engraftment of immature CD34^+^ progenitors [25]. Findings of other studies [26], [27] have positioned PKCζ in the center of chemoattractant and immunoregulator-induced responses. Activation/phosphorylation of PKCζ by HGF is an essential intracellular signaling requirement for growth factor-inducedβ-cell proliferation [28]. Recent reviews also described the involvement of atypical PKCζ/ι in HGF-induced Ras-related C3 botulinum toxin substrate (Rac) activation and membrane ruffling of colony growing epithelial cells [23]. Therefore, we speculated that PKCζ may be involved in HGF-induced CXCR4 expression in breast cancer, and may affect the behaviors of migration and invasion in breast cancer cells.

In the present study, we demonstrated that HGF-induced activation of PKCζ increases CXCR4 expression and the migratory capacity of MDA-MB-436 breast cancer cells. A molecular analysis of these events indicated that augmented CXCR4 expression was regulated by PKCζ activity. The phosphatidylinositol 3 (PI 3)-kinase and protein kinase B (PKB/AKT) pathways were involved in CXCR4 expression and the HGF-induced activation of PKCζ. The functional importance of HGF-induced PKCζ activation in breast cancer metastasis was further demonstrated in a xenograft experiment in which the suppression of PKCζ abrogates HGF-induced metastasis of breast cancer to the lung and liver.

## Results

### HGF/c-Met or CXCR4 expression in breast carcinomas correlated with tumor invasiveness

**Figure 1:**
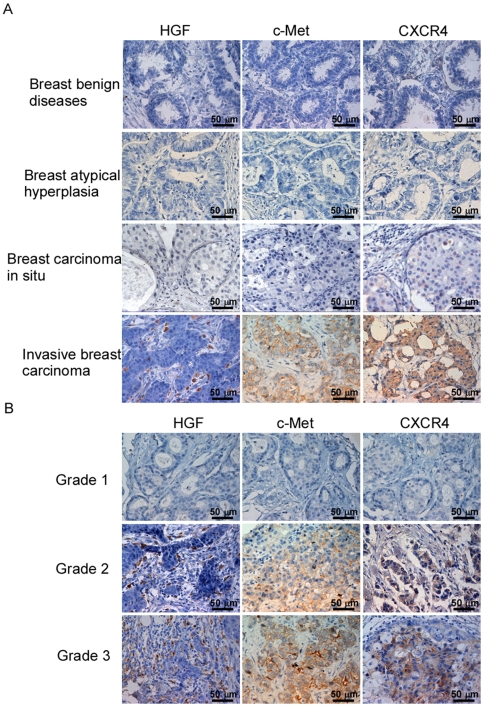
Immunohistochemical staining of HGF, c-Met and CXCR4 in breast cancer specimens. Original magnification, 400×. (A) Representative micrographs of immunohistochemical results for negative HGF, c-Met or CXCR4 staining in breast benign diseases (an atypical hyperplasia or carcinoma in situ) and for positive HGF, c-Met or CXCR4 staining in an invasive breast carcinoma; (B) Representative micrographs of immunohistochemical results for positive HGF, c-Met and CXCR4 staining cell counts, where the intensity of immunohistochemical staining correlates with the histopathological grading of invasive breast carcinomas.

High level of HGF/c-Met expression is considered as a possible indicator of metastasis, earlier recurrence and shortened survival in breast cancer patients [29]. In the tumor microenvironment, HGF regulates the expression of c-Met and CXCR4 through autocrine or paracrine actions [13], [17]. Moreover, the chemokine receptor CXCR4 has been shown to be one of the vital factors for metastasis in breast cancer patients [7]. Initially, we intended to establish whether c-Met and CXCR4 are also expressed on breast cancer cells in clinically resected tissues. To do this, we determined the expression of HGF, c-Met and CXCR4 in breast cancer tissues by immunohistochemistry using corresponding antibodies, which revealed positive immunostaining of HGF, c-Met or CXCR4 in 197 cases of invasive breast carcinoma. In contrast, c-Met^+^ and CXCR4^+^ cells were not found to be present in any of the benign breast tissues with or without atypical epithelial hyperplasia (Figure 1A).

When the c-Met^+^ and CXCR4^+^ cell counts in breast cancer were calculated according to the clinicopathology of these patients (Table S1), the numbers of HGF^+^, c-Met^+^ or CXCR4^+^ cells increased along with the histopathological grading of the tumor (P<0.001) (Figure 1B, Table S1). In addition, c-Met^+^ or CXCR4^+^ cell infiltration appeared to be more intense in those with axillary lymph node (P<0.001) or distal metastasis (P<0.001) (Table S1). These findings linked the enhanced expression of c-Met and CXCR4 to invasiveness and metastasis of breast cancers.

### HGF upregulated CXCR4 protein expression and membrane presentation in human breast cancer cells

**Figure 2:**
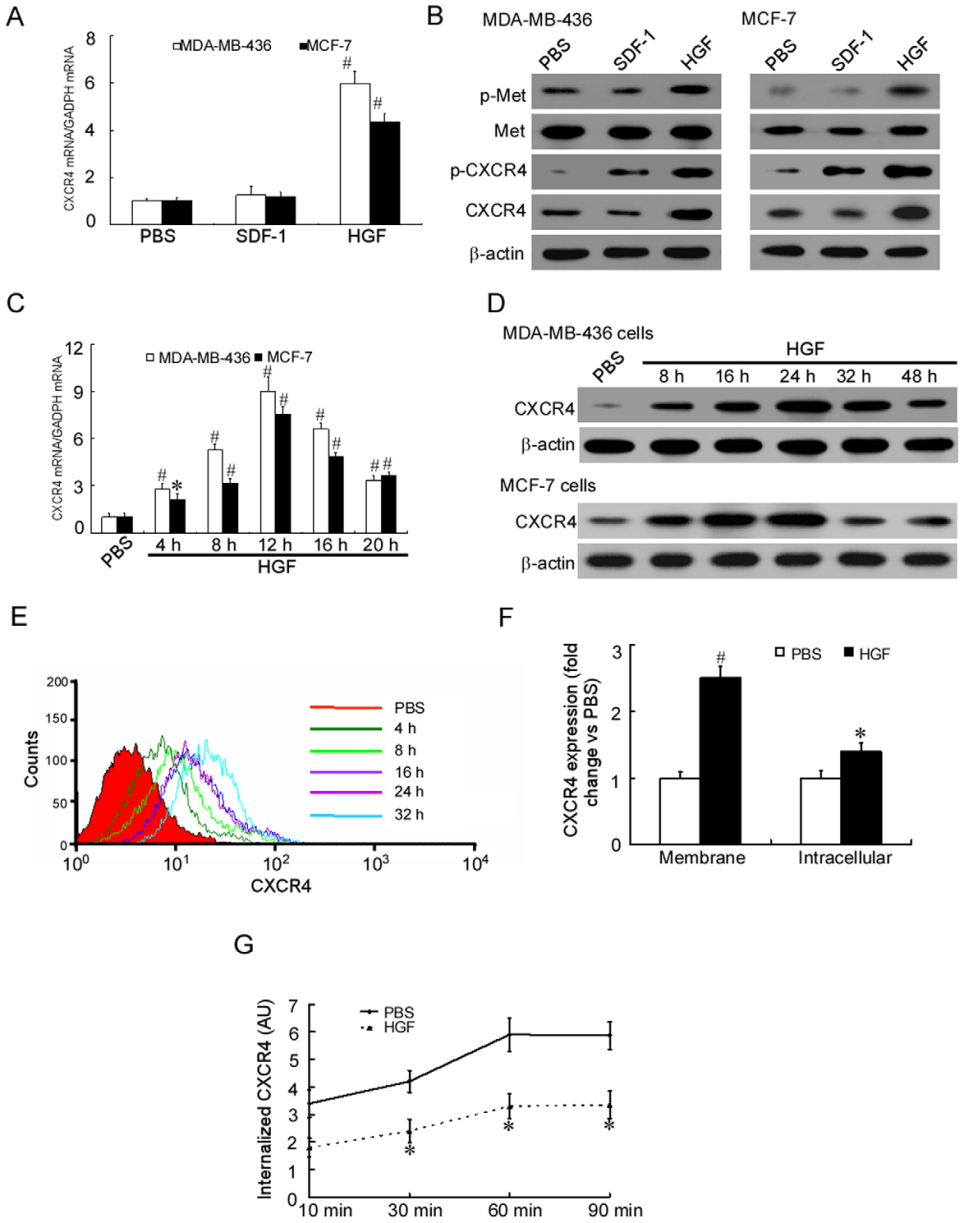
HGF upregulates CXCR4 expression and membrane presentation in human breast cancer cells. (A). qRT-PCR analysis of the total CXCR4 mRNA extracted from HGF- or SDF-1-treated MDA-MB-436 and MCF-7 cells. Results are presented as the mean ± SD of three independent experiments. # P<0.01 as compared to PBS. (B). Western blot analysis of the total protein expression and phosphorylation levels of Met or CXCR4 in MDA-MB-436 and MCF-7 cells cultured for 24 hours with and without the presence of 50 ng/ml HGF or 20 ng/ml SDF-1. The experiment was repeated twice with similar results. A representative study is shown. (C). Time course of the relative extracted CXCR4 mRNA expression in MDA-MB-436 and MCF-7 cells following stimulation with 50 ng/ml HGF. Results are presented as the mean ± SD of three independent experiments. # P<0.01,* P<0.05 as compared to PBS. (D). Western blot analyses of CXCR4 protein expression in HGF-treated MDA-MB-436 and MCF-7 cells. The experiment was repeated three times with similar results. A representative study is shown. (E). Flow cytometric analysis of MDA-MB-436 cells stained for the expression of CXCR4 using a CXCR4 monoclonal antibody and isotype controls. The experiment was repeated three times with similar results. A representative study is shown. (F). Increased membrane and intracellular CXCR4 labeling in MDA-MB-436 cells incubated for 24 hours in the presence of 50 ng/ml HGF compared with untreated cells (PBS) taken as 1. Flow cytometry analysis data (mean±SD of three independent experiments) are shown. # P<0.01,* P<0.05 as compared to PBS. (G). Decreased internalization rate of anti-CXCR4-PE mAb in MDA-MB-436 cells treated for 24 hours with HGF compared with PBS. Results are presented as the mean ± SD of three independent experiments. * P<0.05 as compared to PBS at each time point.

Tumor development involves an intricate set of molecular events driven by different environmental stimuli. Changes in stromal cell-released cytokines (proinflammatory cytokines, chemokines and growth factors) seem to influence the gene expression profile in malignancy and to determine the cell fate [30]. To further study the effect of HGF on CXCR4 expression, MDA-MB-436 and MCF-7 breast cancer cell lines with different grades of malignancy were cultured in the presence or absence of HGF or SDF-1 for 24 hours. Treatment with HGF was found to result in a 2- to 7-fold increase in CXCR4 mRNA and protein expression in the MDA-MB-436 and MCF-7 cells (Figures 2A–2B, Figure S1A). To study the level of Met expression and the effect of HGF on Met expression, MDA-MB-436 and MCF-7 breast cancer cell lines were cultured in the presence or absence of HGF or SDF-1 for 24 hours. Treatment with HGF was found to result in increased Met phosphorylation but not Met expression in the MDA-MB-436 and MCF-7 cells (Figure 2B, Figure S1B).

To study the possible correlation between the patterns of CXCR4 mRNA and protein expression, we evaluated mRNA or protein expression of CXCR4 after HGF treatment by quantitative RT-PCR (qRT-PCR) or immunoblotting (Figures 2C–2D) and flow cytometry (Figure 2E). The level of CXCR4 mRNA increased by approximately 2 to 9 folds between 4 and 12 hours after HGF treatment, and then declined gradually (Figure 2C). The level of CXCR4 protein began to increase by 4 hours, doubled between 8 and 16 hours, and was approximately 2- to 6-fold higher than in the starved cells at 24 hours after HGF treatment (Figures 2D–2E, Figure S1B). The difference in the time course was probably consistent with the time required for protein synthesis, although we cannot exclude the possible involvement of post-transcriptional mechanisms therein. Our study also found that HGF treatment promoted the expression of CXCR4 and led to CXCR4 phosphorylation. In contrast, SDF-1, the ligand of CXCR4, another important cytokine functioning in a different way, was also found to cause CXCR4 phosphorylation but did not increase expression of CXCR4 mRNA and protein in MDA-MB-436 and MCF-7 cells (Figures 2A–2B, Figure S1A). Changes in the membrane and intracellular CXCR4 levels were then comparatively examined in MDA-MB-436 cells incubated for 24 hours with HGF. While cell surface (membrane) expression of the receptor was upregulated by 2.5-fold, only a 1.4-fold increase was found for the level of intracellular CXCR4 (Figure 2F). Furthermore, a time course analysis of surface-bound anti-CXCR4 mAb internalization showed that CXCR4 receptor endocytosis was reduced by 2-fold in HGF-stimulated cells compared with untreated counterparts (Figure 2G), suggesting that in the absence of ligand, HGF stimulation may contribute to longer duration for CXCR4 to be detectable on cell membrane.

### HGF induced CXCR4 expression in a PKCζ-dependent fashion

To investigate the mechanisms underlying the effects of HGF on CXCR4 expression we examined downstream constituents of the HGF-activated pathway in MDA-MB-436 cells. Studies have shown that cell responses to HGF/SF binding involve activation of different functional PKC subspecies that are associated with enhanced cell migration and growth [14], [29]. We thus focused on PKCζ which had recently been implicated in HGF-induced signaling [23].

**Figure 3:**
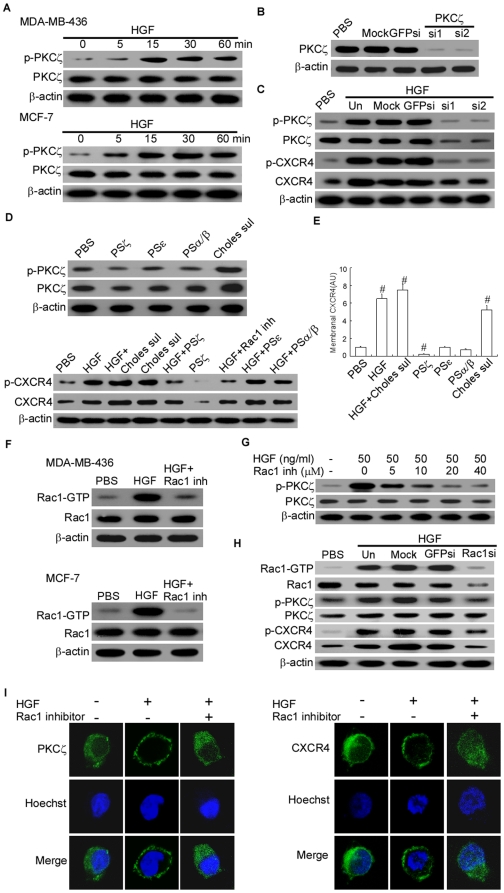
HGF-induced increase in CXCR4 expression depends on PKCζ activity. (A). Time course of relative p-PKCζ levels as determined by immunoblot in MDA-MB-436 and MCF-7 cells following stimulation with 50 ng/ml HGF. The experiment was repeated three times with similar results. A representative study is shown. (B) and (C). 100 µM PKCζ-siRNA1 (si1) or PKCζ-siRNA2 (si2) transient transfections and siRNA-mediated PKCζ protein silencing in MDA-MB-436 cells. p-CXCR4 and p-PKCζ expression levels in MDA-MB-436 cells cultured for 24 hours with 50 ng/ml HGF or/and 100 µM PKCζ-siRNA1 (si1) or PKCζ-siRNA2 (si2). The experiment was repeated three times with similar results. A representative study is shown. (D). Western blot analysis of total CXCR4 expression and p-CXCR4 levels in MDA-MB-436 cells cultured for 24 hours with 50 ng/ml HGF, 2 µM cholesterol sulfate (Choles sul), or PBS. As indicated, 10 µM PS of PKCζ (PSζ), PKCε (PSε), or PKCα/β (PSα/β) or 25 µM NSC23766 (Rac1 inhibitor, Rac1 inh) was used. The experiment was repeated three times with similar results. A representative study is shown. (E). Membrane CXCR4 expression in MDA-MB-436 cells incubated for 24 hours with 50 ng/ml HGF, 2 µM Choles Sulfate, or PBS. Where indicated, 10 µM PS of PSζ, PSε, or PSα/β was used. The flow cytometry analysis data are shown in arbitrary units (AU) normalized to PBS as the mean ± SD of three independent experiments. # P<0.01 as compared to PBS. (F). Western blot analysis of Rac1-GTP and total-Rac1 in MDA-MB-436 and MCF-7 cells treated with 50 ng/ml HGF with or without 25 µM NSC23766. The experiment was repeated three times with similar results. A representative study is shown. (G). Dose-dependent inhibition of HGF-induced p-PKCζ was achieved using NSC23766 in HGF-treated MDA-MB-436 cells; the cells were assayed by western blot. The experiment was repeated three times with similar results. A representative study is shown. (H). Western blot analysis of Rac1, PKCζ and CXCR4 expression in MDA-MB-436 cells cultured for 48 hours with 100 µM Rac1-siRNA. The experiment was repeated three times with similar results. A representative study is shown. (I). Cellular distribution of PKCζ (left panel) and CXCR4 (right panel) before and after HGF (50 ng/ml) or NSC23766 (50 µM) stimulation for 10 minutes. Representative of two independent experiments was shown.

Activation of PKC requires phosphorylation by an upstream kinase which results in translocation to the plasma membrane where PKC can phosphorylate its substrate. To verify whether HGF/SF activates conventional or novel PKC isoforms in MDA-MB-436 cells, we performed western blot analyses of extracts from treated and unstimulated cells, and found that HGF had a major effect on PKCζ activity. As shown in Figure 3A, the level of phosphorylated PKCζ in MDA-MB-436 and MCF-7 cells increased by approximately 3- to 10-fold within 5 to 60 minutes after HGF treatment, in consistency with a hallmark feature of PKCζ activation (Figure 3A, Figure S2A). To obtain an appreciable down-regulation of cellular atypical PKCs, we silenced PKCζ using different combinations of specific siRNAs. This resulted in impairment of HGF-induced expression and phosphorylation of both PKCζ and CXCR4 (Figures 3B–3C, Figures S2B–2C). Addition of the PKCζ inhibitory pseudosubstrate (PSζ) was shown to substantially affect the basal CXCR4 expression and completely abrogated HGF-induced CXCR4 expression in MDA-MB-436 cells (Figures 3D–3E). In contrast, inhibition of other PKC isoenzymes (PKCε and PKCα/β) did not produce any change in CXCR4 expression and phosphorylation (Figure 3D, Figures S2D–2E)). Moreover, after treatment with cholesterol sulfate (a chemical activator of PKCζ [31]), the expression of membrane CXCR4 proteins in MDA-MB-436 cells was significantly enhanced (Figure 3E), which was similar to that in HGF-treated cells.

Members of the Rac small GTPases family have been implicated in the spread and dissociation of the HGF-induced activation of the Cdc42/Rac-regulated p21-activated kinase (PAK) and c-Jun N-terminal kinase to membrane ruffles [32]. An increasing body of evidence indicates that Rac mediates the localization and activation of atypical PKCζ in epithelial cells [23], [33]. Therefore, we examined the involvement of Rac-GTPases in HGF-induced PKCζ activation, and found that treatment with HGF increased Rac1 activity in MDA-MB-436 and MCF-7 cells (Figure 3F, Figure S2F). For an insight into this observation, our test with NSC23766 (a Rac-specific small-molecule inhibitor that targets Rac activation by GEF [34]) demonstrated that the HGF-induced increase in Rac1 activities can be blocked by Rac1 inhibitor, and that the activities of Rac1 was involved in HGF-induced PKCζ phosphorylation. The concentration of Rac1 inhibitor was inversely associated with the level of HGF-induced PKCζ phosphorylation (Figure 3G, Figure S2G). To directly test the role of total Rac activation levels in regulating HGF-induced PKCζ phosphorylation in breast cancer cells, levels of Rac1 were knocked down using RNA interference with small interfering (si)RNA. Reductions in Rac protein levels resulted in proportional changes in Rac activity, and in turn, interfered with PKCζ phosphorylation and expression of CXCR4 (Figure 3H, Figure S2H). Immunocytochemical analysis revealed that NSC23766 completely abrogated the HGF-induced translocation of PKCζ from cytoplasm to cell membrane in MDA-MB-436 cells (Figure 3I), suggesting that functional Rac1 might be required for PKCζ activation by HGF. Importantly, blocking Rac1 activation in MDA-MB-436 cells caused a reduction in membranal and intracellular CXCR4 expression comparable to that of PSζ or PKCζ siRNAs in a manner that was neither synergistic nor additive in the presence of the both inhibitors (Figure 3C–3D, Figure 3I).

This result supported our hypothesis that Rac1 and PKCζ are involved in the same pathway in HGF-stimulated elevation of CXCR4 expression.

### HGF-induced CXCR4 expression was functional and depended on PKCζ for its activity

The role of CXCR4 in cancer depends on whether it is in an activated signaling state. CXCR4 is phosphorylated in response to binding of CXCL12 in a G protein-coupled receptor kinase 2-dependent fashion [35]. Receptor phosphorylation stimulates the interaction between β-arrestin and the carboxy terminus [36]. Phosphorylation of CXCR4 can also occur in response to the activation of other receptors and involve additional kinases such as protein kinase C [37] or tyrosine kinases [12]. To better define the significance of HGF-induced CXCR4 expression, we designed the following experiments. The first set of experiments was based on western blot analysis of MDA-MB-436 cells treated with HGF or SDF-1. The level of phosphorylated CXCR4 was dramatically increased after treatment with either HGF or SDF-1 (Figure 2B, Figure S1A). Cholesterol sulfate increased the level of phosphorylated CXCR4 similarly to as found in HGF-treated cells. Addition of PKCζ inhibitory PSζ was found to substantially affect the expression of phosphorylated CXCR4. In contrast, inhibition of other PKC isoenzymes (PKCε and PKCα/β) produced no change in expression of phosphorylated CXCR4 (Figure 3D, Figure S2E).

**Figure 4:**
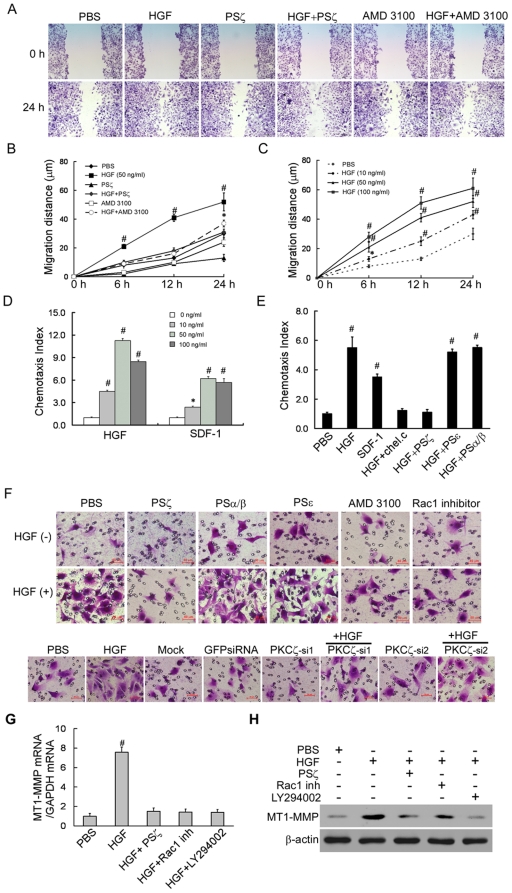
Overexpressed CXCR4 in HGF-stimulated MDA-MB-436 cells is functional. (A–B). Confluent cells were grown in 0.5% FBS medium for 24 hours and were then wounded with a tip. The cells were washed, and the medium was replaced with or without addition of HGF, PSζ peptides or AMD3100. Representative micrographs of the wounds are shown together with the results of the migration quantification. Results are presented as the mean ± SD of 3 independent experiments. # P<0.01 as compared to PBS. Original magnification, 200×. (C).Dose and time-dependent response of HGF-induced MDA-MB-436 cell migration. (D).Dose-dependent response of HGF-induced MDA-MB-436 cell chemotaxis. (E).PKC and CXCR4 regulate HGF-mediated chemotaxis. MDA-MB-436 cells were incubated for 30 minutes with 10 µM chelerythrine chloride, an inhibitor of all PKC, or for 1 hour with 10 µM of PS-α/β, PS-ε, or PSζ peptide. (F). Boyden chamber assays were performed using the SDF-1 ligand for CXCR4 as a chemotactic attractive agent in the lower chamber. AMD3100, PSζ, PSα/β, PSε and NSC23766 (upper) or PKCζ-siRNA (lower) was added to the cell culture for the blocking assay. Data are shown as the mean ± SD of three experiments. A representative study is shown. (G). qRT-PCR analysis of the MT1-MMP mRNA extracted from MDA-MB-436 cells cultured for 24 hours with the indicated agents. Results are presented as the mean ± SD of three independent experiments. # P<0.01 as compared to PBS. (H). Western blot analysis of the total protein expression levels of MT1-MMP in MDA-MB-436 cells cultured for 24 hours with the indicated agents. The experiment was repeated three with similar results. A representative study is shown.

To better understand the steps in which CXCR4 may contribute to HGF-induced invasion, we examined the migration and chemotatic activities of MDA-MB-436 cells in response to HGF stimulation. As shown by the wound healing assays, the migration of HGF-stimulated cell was more efficient compared with PBS-treated cells (Figures 4A–4B), occurring in a dose-dependent manner (Figure 4C). In the presence of 10 µM PSζ or 1 µM AMD3100 (a small-molecule inhibitor of CXCR4), HGF-induced migration was inhibited, which further supported our hypothesis that HGF-stimulated MDA-MB-436 cell migration is mediated by the CXCR4/PKCζ pathway (Figure 4B). We then examined the chemotactic effects of CXCR4 using HGF and SDF-1 gradients. SDF-1 was found to induce the chemotaxis of MDA-MB-436 cells in a dose-dependent manner. HGF-induced chemotaxis also exhibited a typical bell-shaped dose-response curve, and the chemotaxis indexes were 1.5-fold greater than those with SDF-1 induction (Figure 4D–4E). Adding PSζ or chelerythrine chloride (an inhibitor of all PKCs) substantially affected and even abrogated HGF-induced chemotaxis in MDA-MB-436 cells. In contrast, PKCε and PKCα/β, the other PKC isoenzyme inhibitors, had no effect on HGF-induced chemotaxis (Figure 4E). Given that HGF induces CXCR4 expression, and that HGF as well as CXCR4/SDF-1 interaction plays an important role in metastasis, we set to examine whether the SDF-1/CXCR4 interaction contributes to HGF-induced invasive activities. Invasion assays were performed using transwell invasion chambers. When 50 ng/ml of HGF was added to the supplemented medium, there was a 3- to 5-fold increase in the number of invading cells as compared with the PBS-treated cells. These results indicated that HGF enhanced the invasive activity of MDA-MB-436 cells toward SDF-1 in vitro (Figure 4F, Figure S3A–3B). The results showed that HGF did not influence cancer cell viability and proliferation (Figure S3C–3D). These data were consistent with previous report [38]. Moreover, the HGF-induced invasion was inhibited by PKCζ-siRNAs, as well as the effect of PSζ. Conversely, PSε and PSα/β had no effect on HGF-induced invasion (Figure 4F, Figure S3A–3B). In agreement with our findings implicating Rac1 activity in HGF-induced PKCζ activation (Figures 3H–3I), we found that Rac1 inhibited the effect of HGF-induced invasion of MDA-MB 436 cells.

To further define the role of PKCζ in HGF-stimulated processes, we looked at other possible targets of PKCζ that could stimulate metastasis of these cells. The experiments were based on the reports that HGF is a potent activator of membrane type-1 matrix metalloproteinase (MT1-MMP) in breast cancer cells [39], in a PKCζ-dependent manner [40], is a proteolytic enzyme known to be involved in degrading extracellular matrix and assist in cancer invasion and progression. MT1-MMP in coordination with CXCR4 promotes invasion and dissemination of the tumor [41]. Surprisingly, we found that HGF treatment also increased MT1-MMP expression by MDA-MB 436 cells. Inhibition of PKCζ, Rac-1 and phosphatidylinositol 3-kinase by their respective inhibitors PSζ, NSC23766 and LY290042 attenuated MT1-MMP expression in MDA-MB 436 cells (Figure 4G–4H, Figure S3E). These data were consistent with previous reports [42], [43]. Therefore, the proper function of MT1-MMP may depend on both cellular expression and specific membrane localization. Rac1 may be involved in both processes.

These findings implied that up-regulation of CXCR4 by HGF was essential for in vitro migration and invasion via the CXCR4/PKCζ-mediated pathway. HGF induction of Rac1 activation and MT1-MMP production implied that cell migration and proteolysis are two essential processes during tumor invasion and metastasis.

Our data suggested that PKCζ stimulation and CXCR4 expression increased the in-vitro migration and invasion of HGF-treated MDA-MB-436 cells.

### HGF-induced CXCR4 expression was mediated via activation of the PI 3-kinase and AKT signaling modules

**Figure 5:**
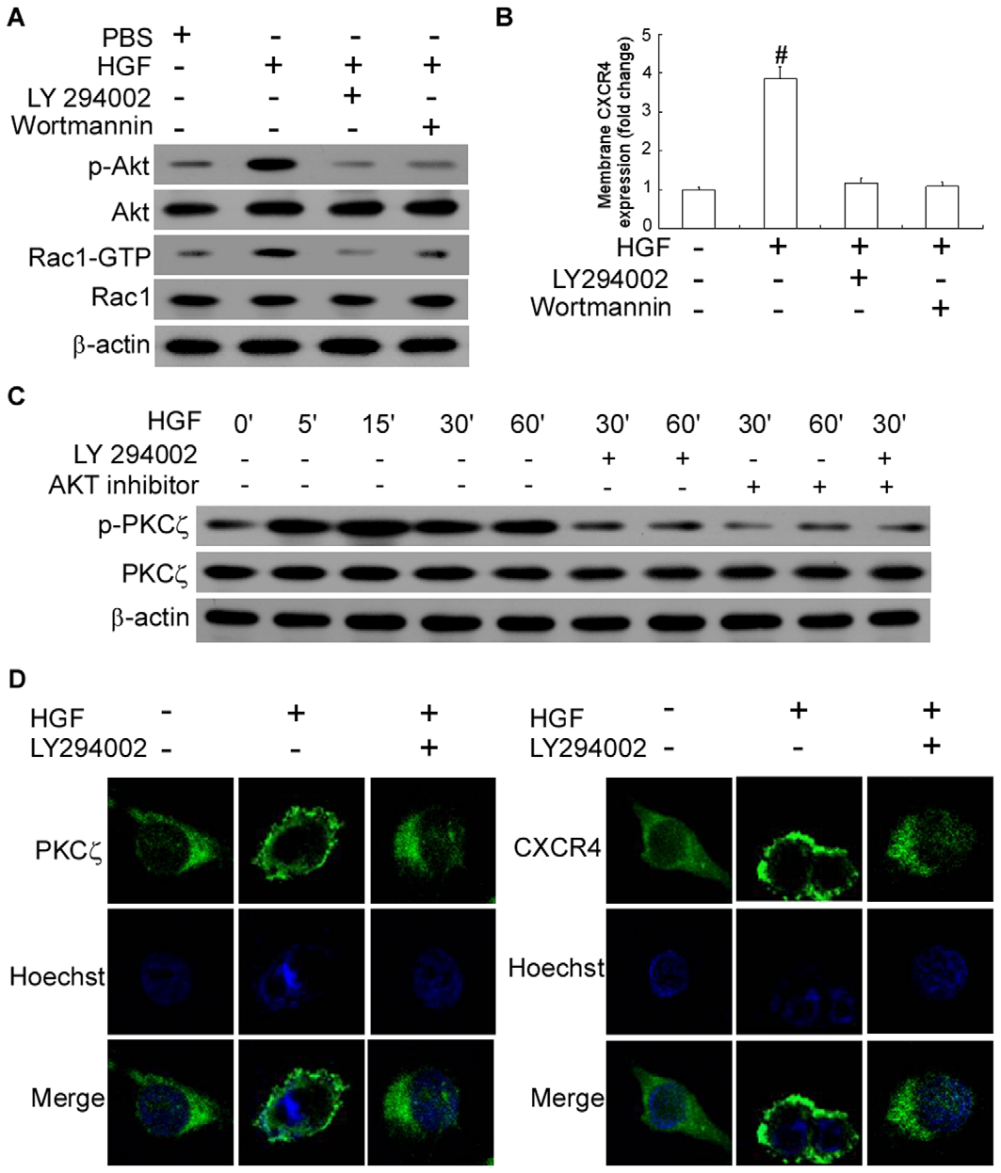
HGF results in PI3K/Akt pathway phosphorylation and activates CXCR4 phosphorylation via PKCζ. (A). Detection of phosphorylated Akt or of the respective total Akt protein expression and Rac1-GTP or total Rac1 expression by western blot analysis. MDA-MB-436 cells were added with PBS or were exposed to HGF/SF (for 10 minutes at 50 ng/ml) after preincubation with the PI3-kinase inhibitors wortmannin (at 100 nM, for 60 min) or LY 294002 (at 10 µM, for 60 min) as indicated. The experiment was repeated twice with similar results. A representative study is shown. (B) Membrane CXCR4 expression in MDA-MB-436 cells cultured in the absence or presence of wortmannin (100 nM) or LY 294002 (50 µM, starting 1 hour prior to HGF/SF treatment) with or without HGF/SF treatment (for 16 hour at 50 ng/ml) as indicated. The flow cytometry analysis data are shown in arbitrary units (AU) as the mean ± SD of three independent experiments. # P<0.01 as compared to PBS. (C) MDA-MB-436 cells were exposed to HGF with or without PI 3-kinase inhibitor LY294002 (30 µM) or Akt inhibitor III (50 µM) for various amounts of time, which resulted in the phosphorylation of PKCζ. Western blotting of total protein were performed in triplicate. A representative study is shown. (D). Cellular distribution of PKCζ and CXCR4 before and after 30 minutes of HGF stimulation. HGF, 50 ng/ml, for 10 minutes; LY294002, 30 µM, for 60 min. The experiment was repeated three times with similar results. A representative study is shown.

In an attempt to identify the pathway leading to PKCζ activation, we investigated the role of phosphatidylinositol triphosphate-kinase (PI 3K) which has been proposed to activate PKCζ [44]. Akt is a pivotal effector immediately downstream of the PI 3K and has been implicated in G protein-coupled receptor-mediated chemotaxis [45]. The HGF-induced Akt activation is reflected by an increase in phosphorylated Akt [46]. To verify whether HGF activates Akt phosphorylation in MDA-MB-436 cells, we performed western blot analyses of extracts from treated and unstimulated cells, and found that HGF had a major effect on Akt activity. Adding PI 3K inhibitors LY294002 and wortmannin was shown to partially prevent the phosphorylation of AKT in MDA-MB-436 cells (Figure 5A, Figure S4A). Furthermore, LY294002 and AKT inhibitor III inhibited PKCζ phosphorylation and membrane CXCR4 expression, which suggested that PKCζ exerted its function downstream of the PI 3-kinases signaling pathway (Figures 5B–5C, Figure S4B). Pretreatment with LY294002 was also found to prevent translocation of PKCζ and CXCR4 to the plasma membrane (Figure 5D), demonstrating that PKCζ activation depended on PI3K activity.

Rac1 reportedly acts as a downstream effector of PI3-kinase in several growth factor-stimulated pathways [47]. Rac1 activation is often dependent on PI3-kinase activity, and inhibitors of PI3-kinase block Rac1 activation. To verify whether HGF activates Rac1 activities in MDA-MB-436 cells and the relationship of Rac1 and PI3K pathway, we performed Rac1 activity assay analyses of extracts from treated and unstimulated cells, and found that HGF can increase Rac1 activities. Adding PI 3K inhibitors LY294002 and wortmannin was shown to partially decrease the activity of Rac1 in MDA-MB-436 cells (Figure 5A, Figure S4A). Our studies conformed that PI3-kinase inhibitors can suppress Rac1 activities, which further suggested that the PI3-kinase regulate the activity of small GTPases Rac1 [48].

These findings implied that functional CXCR4 expression induced by HGF might involve a pathway of activation of the PI 3K/Akt and PKCζ, and that signals stimulated by HGF passed from PI 3K/Akt to Rac1 and then to PKCζ.

### HGF enhanced CXCR4 expression via PKCζ and promoted the invasion and metastasis of breast cancers in vivo

**Figure 6:**
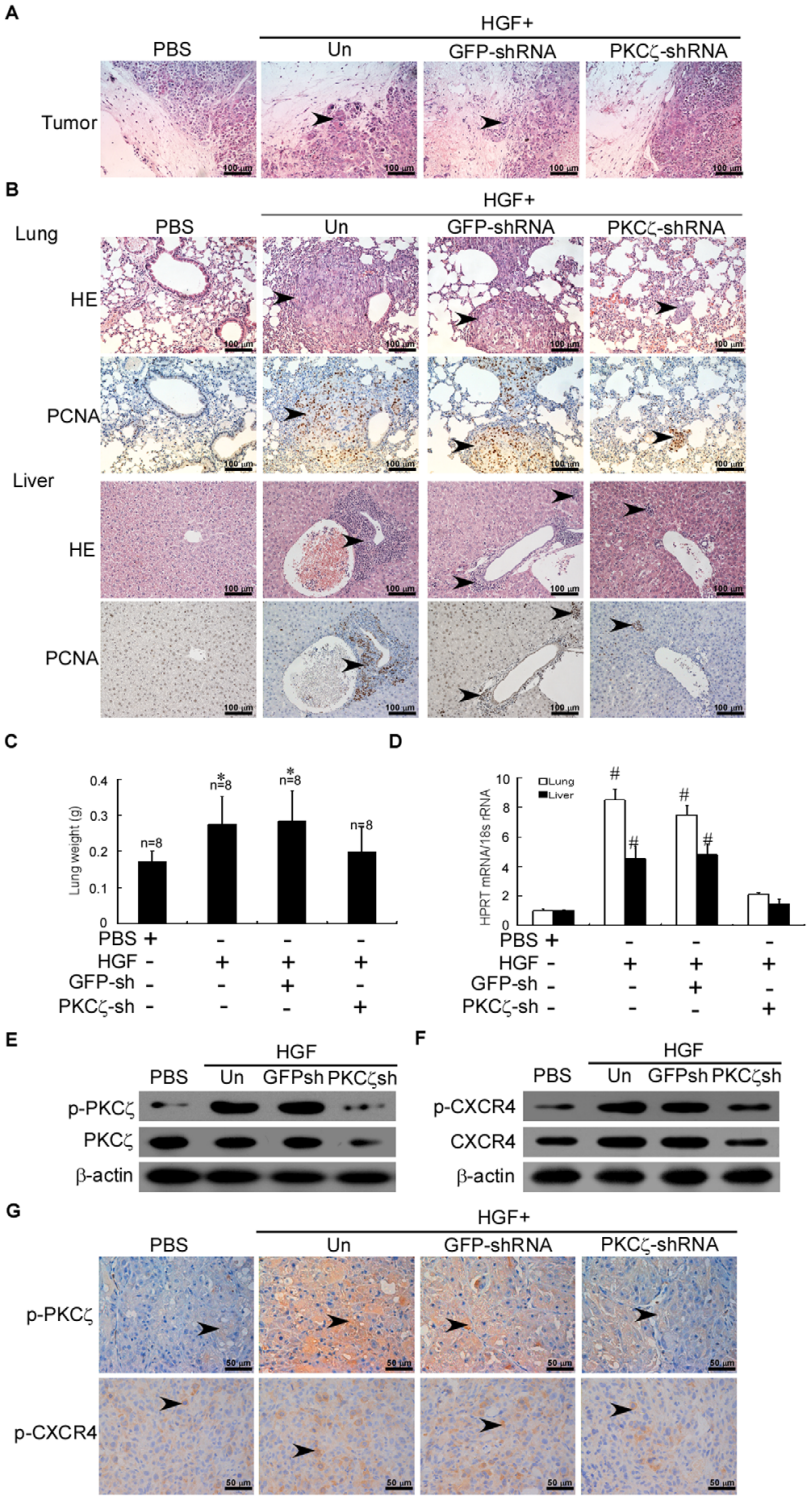
HGF enhances CXCR4 expression via PKCζ and promotes the invasion and metastasis of breast cancers in BALB/c-nu mice. (A) Micrographs of H&E-stained xenografts demonstrating the presence or absence of margin invasion by the MDA-MB-436 tumors untransduced (Un) or transduced with GFP-shRNA or PKCζ-shRNA (original magnification, 200×). (B) Representative micrographs of lung (upper) or liver (lower) tissue sections with H&E staining and PCNA immunohistochemical staining (original magnification, 200×). Each group from two independent experiments. (C) Mean ± SD wet lung weight in tumor-bearing mice. The number of mice in each group is indicated. * P<0.05 as compared to PBS-treated mice. (D) Expression of human HPRT mRNA relative to mouse 18S rRNA as determined by qRT-PCR in the lungs and livers of tumor-bearing mice. Data were normalized to PBS-treated mice. # P<0.01 as compared to PBS-treated mice. (E–F) Immunoblot of total PKCζ and p-PKCζ or total CXCR4 and p-CXCR4 protein, respectively, in breast tumor xenografts in female nude mice that were inoculated in the mammary fat pads with MDA-MB-436 cells treated as indicated. The experiment was repeated twice with similar results. A representative study is shown. (G) Representative microphotos of immunohistochemical results for p-PKCζ (middle) or p-CXCR4 (lower) expression in MDA-MB-436 tumors (original magnification, 400×).

To further examine the effects of HGF-induced CXCR4 expression via PKCζ on breast cancer invasion and metastasis in vivo, we inoculated the mammary fat pads of athymic nude mice with MDA-MB-436 cells. Cancer metastasis to the lung and liver of the mice was evaluated when the xenografts reached 1.5 cm in diameter. Biweekly intratumoral injection with HGF at a dosage of 30 µg/kg for 4 consecutive weeks enhanced the penetration of MDA-MB-436 cancer cells into the adjacent normal tissues, which was not seen with PBS injection (Figure 6A).

Moreover, intratumoral injection of HGF conspicuously increased the number of mice with lung and liver metastasis (Table S2) but not the size of breast cancer xenografts (Figure S5). As observed with hematoxylin and eosin (H&E) and PCNA staining, HGF injection led to more intense infiltration of cancer cells into the lung and liver of MDA-MB-436 xenograft-bearing mice than did the PBS injection (Figure 6B). The mean wet lung weight in HGF-injected tumor-bearing mice was greater as compared with those injected with PBS or PKCζ-shRNA (Figure 6C). This result was further quantified by qRT-PCR for human HPRT, which demonstrated that HGF increased the number of metastatic breast cancer cells in the lung and liver of MDA-MB-436 xenograft-bearing mice by about 7- and 4-fold, respectively, as compared with PBS injection (Figure 6D). Collectively, these data suggested that HGF enhanced the metastasis of breast cancer xenografts to the lung and liver.

HGF injection failed to enhance the peritumoral penetration of MDA-MB-436 xenografts infected with PKCζ-shRNA but not GFP-shRNA (Figure 6A, Figure S8). Moreover, the prometastatic effect of HGF on MDA-MB-436 xenografts was tremendously alleviated by infection with PKCζ-shRNA but not GFP-shRNA (Table S2). Immunoblot and immunohistochemistry with the anti-phospho-PKCζ or anti-phospho-CXCR4 antibody further confirmed that PKCζ-shRNA reduced the expression of phosphorylated PKCζ and HGF-induced phosphorylated CXCR4 in cancer cells of the MDA-MB-436 xenografts (Figures 6E–6G, Figures S6,S7). These findings shed light on the HGF-enhanced invasiveness of cancer cells via PKCζ in tumor-bearing mice.

## Discussion

The present study addressed the molecular aspects of the regulation of CXCR4 in human breast cancer cells. Our results showed that CXCR4 expression was increased and PKCζ activity involved in the HGF-mediated effects. We demonstrated that in breast cancer cells, PKCζ was directly stimulated by HGF-activated signaling. Furthermore, blocking PKCζ activity in MDA-MB-436 cells was found to significantly abrogate membrane CXCR4 expression. Importantly, inhibition of several other PKC isoenzymes did not produce any effect on MDA-MB-436 cells in terms of CXCR4 expression as well as migration, chemotaxis and invasion of the cells.

In this study, the increase in CXCR4 expression in HGF-stimulated cells may be arising from enhanced transcription, but more importantly, from the prolonged time of membrane receptor exposure. Previous studies and our results on anti-CXCR4 Ab internalization suggested that HGF treatment led to a reduction in CXCR4 endocytosis. Consistent with published data, we also noted that HGF-activated CXCR4 expression was attenuated in the presence of PKCζ inhibitor. The level of CXCR4 receptor expression may affect the strength of receptor signaling and determine the pattern of receptor-mediated effects. Protein phosphorylation is the most prevalent posttranslational modification and plays a major role in regulating protein function [49]. More importantly, as one of the earliest events in regulating G protein-coupled receptor (GPCR) signaling, phosphorylation initiates a process known as desensitization. Recent studies demonstrated that site-specific phosphorylation of CXCR4 is dynamically regulated by multiple kinases, which results in both positive and negative modulation of CXCR4 signaling [10]. The present study showed that CXCR4 phosphorylation was induced by HGF treatment in MDA-MB-436 or MCF-7 cells, and was attenuated by inhibition with PKCζ. Therefore, we suggested that CXCR4 expression and CXCR4 phosphorylation correlate with PKCζ in the HGF-mediated effects.

Attempts to elucidate the molecular mechanisms underlying HGF-induced CXCR4 expression led to our interest in the role of Rac1, a member of the Ras superfamily of small guanosine triphosphatases (GTPases) that act as molecular switches to control cytoskeletal rearrangements and cell growth [50]. Indeed, the Rac family proteins, which are effectors of HGF signaling, are critical regulators of chemokine-induced integrin activation and motility [51]. Chianale and coworkers [23] have shown that in the presence of diacylglycerol kinase α, HGF promotes dissociation (from Rac/RhoGDI complex) and activation of Rac, which in turn promotes cytoplasm-to-membrane translocation and phosphorylation of PKCζ. Meanwhile, activated Rac-1 interacts with CXCR4 in lipid rafts on the cell surface resulting in enhanced sensitivity and responsiveness of hematopoietic cells to an SDF-1 gradient [52]. Other findings [53] indicated that binding of Rac1 to Part6 may lead to PKCζ phosphorylation. Consistent with these studies, our results confirmed that: (1) HGF can promote Rac activation, which further promotes the cytoplasm-to-membrane translocation and phosphorylation of PKCζ; (2) HGF stimulation contribute to longer duration for CXCR4 on cell membrane and reduce the CXCR4 endocytosis caused by CXCR4 antibodies. (3) NSC23766, a specific Rac1 inhibitor, can inhibit the activation of Rac1 and impair the HGF-induced PKCζ phosphorylation; and (4) Blocking PKCζ phosphorylation subsequently interfered with the membrane expression and phosphorylation of CXCR4. Therefore we hypothesized that HGF induces Rac recruitment at ruffling sites, which may prolong the membrane expression of CXCR4/Rac-1, and reduce the CXCR4 endocytosis caused by chemoattractants and/or CXCR4 antibodies, thereby stimulating the chemotaxis and migration of tumor cells. Subsequently, our findings suggested that functional Rac1 is required for HGF-induced PKCζ activation. Rac1 and PKCζ constitute a signaling pathway of HGF-induced CXCR4 expression.

Thus far, a further elucidation is needed for the molecular mechanism that governs HGF-induced PKCζ activation and CXCR4 expression. We know that HGF triggers motility dependently on the activation of several intracellular molecular pathways. The roles of PI 3-kinases [54], [55], Ras and 41/43 kDa mitogen-activated protein kinase [56] in HGF-mediated cell scattering activity have also been established. PI 3K exists in a wide variety of cells and could be activated by most receptor tyrosine kinases, G protein-coupled receptors and non-receptor tyrosine kinase relative receptors. Akt is one of the downstream signals of the PI 3K pathway and is also a serine/threonine kinase. Recently, PI 3-kinase has been shown to mediate lymphocyte adhesion and migration by regulating Rho and PKCζ [57]. In order to investigate whether PI3K/Akt were involved in the effects of HGF-induced CXCR4 expression in MDA-MB 436 cells, we examined the changes of CXCR4 expression after HGF-induced PKCζ activation by PI3K/Akt inhibitors. Western blot assays and immunofluorescent staining data suggested that PI 3K/Akt are required for PKCζ activation and CXCR4 expression. Furthermore, Rac1 activation is also dependent on PI3-kinase activity. The lack of complete inhibition may indicate that activation of a small percentage of Akt/PKB is sufficient for chemotaxis, or that an unidentified signaling molecule downstream of the PI 3K shares a partially redundant function with Akt/PKB. Those results also indicated that the mechanism of HGF-induced CXCR4 expression in the MDA-MB-436 breast cancer cell line involved a pathway of activation of the PKCζ and PI 3K/Akt, and that signals stimulated by HGF passed on from PI 3K/Akt to Rac1 and then to PKCζ. These were further supported by the fact that multiple chemical inhibitors and PSζ can block signal transmission.

MT1-MMP, also known as MMP-14, has a single transmembrane domain and is an integral membrane protein with an extracellular catalytic domain suggested to be a key enzyme in tumor metastasis and angiogenesis. Numerous studies implied that MT1-MMP in breast tumors are correlated with the outcome of patients with breast cancer [39]. Reduction of MT1-MMP from the breast cancer cells resulted in significant reduction of in vitro invasiveness and loss of response to an invasion stimulus. An association between MT1-MMP and the PKCζ activation has been reported. PKCζ may be responsible for the increased binding of Sp1 to MT1-MMP promoter site in shear-stressed endothelial cell (EC) and thus the induction of MT1-MMP protein [40]. MT1-MMP in coordination with CXCR4 promotes invasion and dissemination of the tumor [41]. HGF has also been shown to actively promote production of MT1-MMP in MDA-MB 231 cells, which further activated MMP2 and enhanced the invasiveness of breast cancer cells [39]. Thus, we hypothesized that PKCζ may be involved in the signaling pathway that mediates MT1- MMP expression in cells exposed to HGF. Our data were consistent with the requirement of PKCζ phosphorylation and activation for Rac1 increased MT1-MMP expression by MDA-MB 436 cells. Inhibition of PKCζ, Rac-1 and phosphatidylinositol 3-kinase by their respective inhibitors PSζ, NSC23766 and LY290042 attenuated MT1-MMP expression in MDA-MB 436 cells. Therefore, the proper function of MT1-MMP may depend on both cellular expression and specific membrane localization. This study demonstrated that PKCζ is critical to regulation of MT1-MMP expression. Activation of PKCζ is also required in both EGFR- and chemokine receptor-mediated chemotaxis, suggesting the use of PKCζ as a promising target for more potent anti-metastasis therapeutic strategies. Because PKCζ-deficient mice are grossly normal, the pharmacologic inhibition of this enzyme should not be lethal to the hosts [57]. The identification of PKCζ as a convergence point of EGFR- and chemokine receptor-mediated chemotaxis provided us with a potential novel target for anticancer drugs. We postulated that blocking PKCζ may completely impair the chemotactic activities of some cancer cells and result in reduced in tumor invasion and spreading.

Taken together, the results of our study provided new insights to molecular regulation of CXCR4 and to certain distinct regulatory molecules that can be targeted to modulate the CXCR4 signaling in breast cancer.

## Materials and Methods

### Patients and tissue samples

Samples of primary ductal carcinoma of the breast were obtained from 197 female patients (median age 43.7 years, range 23–75) at the Sun Yat-Sen Memorial Hospital, Sun Yat-Sen University, from January 2002 to October 2007. Both the pathological diagnosis and estrogen receptor (ER)/Her2 status were verified independently by two pathologists. Patients with invasive carcinomas other than ductal carcinomas in-situ (DCIS) underwent 6 cycles of postoperative adjuvant chemotherapy with the FAC regimen (500 mg/m^2^ 5-fluorouracil, 50 mg/m^2^ doxorubicin and 500 mg/m^2^ cyclophosphamide). Patients with ER+ tumors subsequently underwent endocrine therapy according to the NCCN guidelines. Distal metastasis was not found in these patients upon diagnosis but was identified in 45 cases during the postoperative follow-up. Additionally, benign breast tissue samples were collected from 20 cases of benign diseases of the breast with or without atypical epithelial hyperplasia. All samples were collected with patient informed consent according to the Internal Review and Ethics Boards of the Sun Yat-Sen Memorial Hospital of Sun Yat-Sen University.

### Cell cultures

The human breast cancer cell lines MDA-MB-436 and MCF-7 were obtained from the American Type Culture Collection. They were cultured in L-15 or RPMI 1640 (Gibco, Birmingham, USA) with 10% fetal bovine serum (FBS) (Gibco, Birmingham, USA). Chemotaxis chambers and membranes were purchased from Costar.

### Wound healing assay

The wound healing assay was mainly performed as described previously [53] with a slight modification. MDA-MB-436 breast cancer cells were grown were grown to confluence in 6-well tissue culture plates. The cells were pretreated with mitomycin (10 mg/ml) for 2 h to block proliferation and then cell-free area introduced by scratching with a pipette tip. Cancer cells were allowed for a 24-hour recovery period to close the wound and were photographed using phase-contrast microscopy.

### Chemotaxis assays

The chemotaxis assays were performed as described by the manufacturer (Costar, Corning, NY, USA). Briefly, MDA-MB-436 cells (5×10^5^ cells/ml) were pretreated with mitomycin (10 mg/ml) for 2 h to block proliferation and then suspended in medium (L-15, 0.1% bovine serum albumin, and 25 mM HEPES) were added to the upper chamber. The two chambers were separated by a 10-µm filter pretreated with 0.001% fibronectin in L-15 at 4°C overnight. The chambers were incubated in 5% CO_2_ at 37°C for 12 hours. The filter was then washed, fixed, and stained. The number of migrating cells was counted in three high-power fields (200×). In the inhibitor assay, MDA-MB-436 cells were pretreated with inhibitors at the indicated concentration for 60 minutes before HGF (Sigma, St. Louis, USA) were added at 37°C and then loaded into the upper chamber.

### Boyden chamber assay

Invasion of breast cancer cells was examined using 24-well Boyden chambers (Costar, Corning, NY, USA) with 8-µM inserts coated with fibronectin (Roche, Mannheim, Germany) and matrigel (BD Biosciences, USA). The lower chamber was filled with L-15/BSA medium containing 10 ng/ml SDF. MDA-MB-436 cells (5×10^4^ cells/well) were pretreated with mitomycin (10 mg/ml) for 2 h to block proliferation and then were plated on the inserts with, or without HGF/SF and cultured at 37°C in the upper chambers. After 8 hours, invading cells that crossed the inserts were stained with viola crystalline (0.005%, Sigma, St. Louis, USA) and were counted as cells per field of view using phase-contrast microscopy.

### Flow cytometry of membrane and intracellular CXCR4 expression

Membrane CXCR4 expression was determined by flow cytometry (BD, CA, USA) as described previously [11] using anti-human CXCR4 monclonal antibody (clone 12G5) (Santa Cruz, CA, USA), followed by secondary FITC-conjugated donkey anti-mouse IgG. Isotype matched IgG2a was used as control, showing background labeling with no differences between the treatments. Ratios of mean values of Ab labeled cells to secondary IgG only control samples were calculated using Cell Quest software (BD, CA, USA), and the results were expressed in arbitrary units (AU). Flow cytometry analysis with additional anti CXCR4 mAbs, clones 6H8 and 1D9, demonstrated similar to 12G5 increase following HGF treatment. For the analysis of intracellular CXCR4 levels, MDA-MB-436 cells were immunolabeled with anti-human CXCR4 mAb after blocking the membrane receptors by a 1-hour incubation with 10 µg/ml non-conjugated 12G5 mAb. Membrane CXCR4 internalization was assayed as previously described: MDA-MB-436 cells were pre-incubated for 30 minutes at 4°C with 10 µg/ml of anti human CXCR4-PE Ab, transferred to 37°C for the indicated times, and then washed for 2 minutes on ice at acidic (pH2.2) buffer to remove the surface-bound Ab. The relative proportion (%) of internalized receptor (labeling after acid wash) with respect to total fluorescence intensity was determined by flow cytometry.

### CXCR4 and protein kinase C ζ translocation assay

Cells were cultured for 48 hours before the experiment. They were then starved in binding medium for 3 hours, followed by stimulation with 50 ng/ml HGF at 37°C for 10 minutes before fixation with 4% formaldehyde. As the inhibitor control, cells were treated with 50 µM NSC23766 (Santa Cruz, CA, USA) at 37°C for 1 hour before HGF stimulation. Cells were then permeabilized with 0.2% Triton X-100 in buffer (10 mM HEPES, 20 mM KH_2_PO_4_, 5 mM EGTA, 2 mM MgCl_2_, PBS, pH 6.8), stained with polyclonal antibodies against various PKC isotypes and probed with FITC-labeled goat anti-rabbit antibody. CXCR4 was labeled with 10 µg/ml anti-human CXCR4 mAb. Cells were visualized using a Zeiss LSM 710 inverted fluorescent confocal microscope (Carl Zeiss, Inc, Germany).

### qRT-PCR

qRT-PCR was performed using an ABI Prism 7000 System (Applied Biosystems, Waltham, MA, USA). SYBR Green was used to detect the PCR products. All reactions were performed in triplicate in a 20-µl reaction volume. Primers for human CXCR4, MT1-MMP, GAPDH, HPRT and mouse 18S rRNA were obtained from Takara (Table S3). PCR amplification consisted of an initial denaturation step at 95°C for 5 min, followed by 40 cycles of PCR at 95°C for 20 s, 60°C for 30 s. Standard curves were generated, and the relative amount of target gene mRNA was normalized to GAPDH mRNA. Specificity was verified by melt curve analysis and agarose gel electrophoresis. To quantify cancer metastasis in mouse lungs and livers, qRT-PCR for human hypoxanthine-guanine phosphoribosyltransferase (hHPRT) was performed on TRIzol-isolated total RNA using the primers described for hHPRT and mouse 18S rRNA. After reverse transcription for 45 min at 42°C and Taq activation for 3 min at 95°C, 40 cycles of PCR at 95°C for 12 s and 60°C for 15 s were performed. The relative amount of hHPRT mRNA was normalized to mouse 18S rRNA.

### Transfections

Transient transfections and siRNA-mediated protein silencing in MDA-MB-436 cells were performed using Lipofectamine 2000 reagent (Invitrogen, Carlsbad, CA, USA) according to the manufacturer's instructions. siRNAs were chemically synthesized as double-stranded RNA (GenePharma Co., Ltd., Shanghai, China). siRNAs against canine PKCζ [23]or Rac1 [58] have been described as follows: PKCζ-siRNA 1 sense: 5′-GGCUGUUCCUGGUCAUAGA-3′; antisense: 5′-UCUAUGACCAGGAACAGCC-3′. PKCζ-siRNA 2 sense: 5′-GGUGCACACUUUCCACAGA-3′; antisense: 5′-UCUGUGGAAAGUGUGCACC-3′. Rac1-siRNA sense: 5′-GAGGCCUCAAGACAGUGUUUGACGA-3′; Rac1-siRNA antisense: 5′-UCGUCAAACACUGUCUUGAGGCCUC-3′. GFP-siRNA (GenePharma Co., Ltd., Shanghai, China) was used as negative control.

### Western blotting assay

Western blotting was carried out as described previously [59]. Briefly, protein extracts were resolved by 10% SDS-PAGE, transferred to PVDF membranes, and probed with antibodies against human CXCR4 (Abcam, Cambridge, MA, USA), phosporylated CXCR4 (p-CXCR4) (Abcam, Cambridge, MA, USA), PKCζ (Bioworld, Louis Park, MN, USA), phosporylated PKCζ (p-PKCζ) (Bioworld, Louis Park, MN, USA), Akt/PKB (Santa Cruz, CA, USA), phosporylated Akt/PKB (Santa Cruz, CA, USA), Rac1 (Santa Cruz, CA, USA), Met (Bioworld, Louis Park, MN, USA), phosporylated Met (p-Met) (Bioworld, Louis Park, MN, USA), MT1-MMP(Abcam, Cambridge, MA, USA) and visualized by an enhanced chemiluminescence assay (Pierce).

### Rac activation assay

Rac1 activity was measured using the rac1 activation assay kit (Upstate Biotechnology, NY, USA) with slight modification. Briefly, whole-protein extracts were immunoprecipitated with protein binding domain of p21 activation kinase-1 (PAK1-PBD). PAK1-PBD only binds to activated forms of rac1 and cdc42. Immunoprecipitated proteins were separated in SDS-PAGE (10%) and blotted with anti-rac1 (Santa Cruz BiotechnologyInc., CA, USA).

### Immunohistochemistry

For immunohistochemistry analysis, anti-p-PKCζ monoclonal antibody or anti-p-CXCR4 polyclonal antibody was used as the primary antibody for overnight incubation at 4°C. The sections were subsequently treated with secondary antibody, followed by further incubation with the streptavidin-horseradish peroxidase complex. Diaminobenzidine was used as a chromogen, and sections were lightly counterstained with hematoxylin. The percentage of p-PKCζ- and p-CXCR4-positive tumor cells was calculated by counting 1,000 tumor cells.

### Proliferation and Cell viability assay

MCF-7 cells and MDA-MB 436 cells (3×10^5^/100 µl) were cultured in serum-free medium with HGF (50 ng/ml) and/or PS-ζ peptides (10 µM), AMD3100 (1 µM), NSC23766 (25 µM). After 3 days, [^3^H]-thymidine (1 µCi/well; Amersham Biosciences) was added, and the cells were cultured for an additional 16 hours. Then the cells were harvested onto fiberglass filters, and detected for their radioactivity in a Matrix-96 direct β counter (Packard Instrument Co.). Cell viability was determined by the MTS colorimetric assay using tetrazolium reagent. MCF-7 cells and MDA-MB 436 cells (5×10^3^/500 µl) were cultured in serum-free RPMI with indicated agents. After 4 days, 20 µl of MTS solution were added to each well and incubated at 37°C for 2 hours. Absorbance at 490 nm, which is directly proportional to the number of living cells, was measured on a 96-well plate reader. Data are expressed as a percentage of the untreated cells cultivated under the same conditions.

### Tumor xenografts

Four groups of eight female BALB/c-nu mice each were introduced to the experiment. MDA-MB-436 (2×10^6^) breast cancer cells that were untransfected or transfected with GFP-shRNA or PKCζ-shRNA were inoculated into the mammary fat pads of female BALB/c-nu mice. When the xenografts were palpable (approximately 5 mm in diameter), intratumoral injection of PBS or 30 µg/kg HGF was performed biweekly for four consecutive weeks. Tumor growth was evaluated by monitoring the tumor volume (TV = length×width^2^×0.5) every three days for eight weeks. The animals were sacrificed when the xenografts reached 1.5 cm in diameter, and the mouse tumor xenografts, lungs and livers were harvested for further evaluation. Cryosections (4 µm) of the harvested organs were stained with hematoxylin and eosin (HE) for histological assessment, and total RNA was extracted for qRT-PCR analysis of human HPRT mRNA expression.

### Statistics

All *in vitro* experiments were performed either in triplicate or in quintuplicate. Standard software 13.0 (SPSS, Chicago, IL) was used for statistical analyses. Measurement data were presented as mean ± standard deviation (SD). Statistical analysis was performed by one-way analysis of variance (ANOVA). The differences between the means were tested by an independent sample t-test or Bonferroni's multiple comparison t-test. The *χ* ^2^ test was used to compare percentages. The significance level used was *P*<0.05.

## Supporting Information

Figure S1 **HGF upregulates CXCR4 expression and membrane presentation in human breast cancer cells.** (A). Western blotting analysis revealed that the protein levels of CXCR4 and p-CXCR4 treated with HGF were higher than those treated with SDF in MDA-MB- 436 (left panel) and MCF-7 cells (right panel). Data are shown in arbitrary units (AU) normalized to PBS as the mean ± SD of three independent experiments. # P<0.01 as compared to PBS. (B). Western blotting analysis revealed that the protein levels of Met and p-Met treated with HGF were higher than those treated with SDF in MDA-MB- 436 (left panel) and MCF-7 cells (right panel). Data are shown in arbitrary units (AU) normalized to PBS as the mean ± SD of three independent experiments. # P<0.01 as compared to PBS. (C).Time course of relative CXCR4 content in two breast cancer cells following 50 ng/ml HGF stimulation. Data are shown in arbitrary units (AU) normalized to PBS as the mean ± SD of three independent experiments. # P<0.01 as compared to PBS.
(TIF)

Figure S2 **HGF-induced increase in CXCR4 expression depends on PKCζ activity.** (A). Time course of relative p-PKCζ levels as determined by immunoblot in MDA-MB-436 (left panel) and MCF-7 cells(right panel) following stimulation with 50 ng/ml HGF. Data are shown in arbitrary units (AU) normalized to PBS as the mean ± SD of three independent experiments. # P<0.01, *P<0.05 as compared to PBS. (B).100 µM PKCζ-siRNA1 (si1) or PKCζ-siRNA2 (si2) transient transfections and siRNA-mediated PKCζ protein silencing in MDA-MB-436 cells. Data are shown in arbitrary units (AU) normalized to PBS as the mean ± SD of three independent experiments. # P<0.01 as compared to PBS. (C). Western blot analysis of PKCζ(left panel) and CXCR4(right panel)expression levels in MDA-MB-436 cells cultured for 24 hours with 50 ng/ml HGF or/and 100 µM PKCζ-siRNA1 (si1) or PKCζ-siRNA2 (si2). Data are shown in arbitrary units (AU) normalized to PBS as the mean ± SD of three independent experiments. # P<0.01 as compared to PBS. (D) and (E). Western blot analysis of total CXCR4 expression and p-CXCR4 levels in MDA-MB-436 cells cultured for 24 hours with 50 ng/ml HGF, 2 µM cholesterol sulfate (Choles sul), or PBS. As indicated, 10 µM PS of PKCζ (PSζ), PKCε (PSε), or PKCα/β (PSα/β) or 25 µM NSC23766 was used. Data are shown in arbitrary units (AU) normalized to PBS as the mean ± SD of three independent experiments. # P<0.01*P<0.05 as compared to PBS. (F). Western blot analysis of Rac1-GTP and total-Rac1 in MDA-MB-436 and MCF-7 cells treated with 50 ng/ml HGF with or without 25 µM NSC23766. The experiment was repeated three times with similar results. A representative study is shown. # P<0.01 as compared to PBS. (G). Dose-dependent inhibition of HGF-induced p-PKCζ was achieved using NSC23766 in HGF-treated MDA-MB-436 cells; the cells were assayed by Western blot. Data are shown in arbitrary units (AU) normalized to PBS as the mean ± SD of three independent experiments. # P<0.01 as compared to PBS. (H). Western blot analysis of Rac1, PKCζ and CXCR4 expression in MDA-MB-436 cells treated with 100 µM Rac1-siRNA for 48 hours. Data are shown in arbitrary units (AU) normalized to PBS as the mean ± SD of three independent experiments. # indicates P<0.01 as compared to PBS.
(TIF)

Figure S3 **Overexpressed CXCR4 in HGF-stimulated MDA-MB-436 cells is functional.** (A–B). MDA-MB-436 cells were treated with indicated agents determined by Boyden chamber assays. Cells were counted in triplicate wells and in three identical experiments. Data are shown in invasion index normalized to PBS as the mean ± SD of three independent experiments. # P<0.01 as compared to PBS. (C). Effect of HGF on breast cancer cell proliferation. MDA-MB 436 and MCF-7 cell lines were cultured for 3 days in serum-free medium with HGF (50 ng/ml) and/or other indicated agents, [^3^H]-thymidine (1 µCi/well; Amersham Biosciences) was added for an additional 16 hours. Cells were harvested onto fiberglass filters, and radioactivity was detected in a Matrix-96 direct β counter (Packard Instrument Co.) Results shown are representative of three independent experiments. (D). For the MDA-MB 436 and MCF-7 cells were cultured in the presence of HGF and/or other indicated agents for 4 days. Cell viability was determined by MTS assay and reported as a percent of untreated cells. Results shown are representative of three independent experiments. (E). Western blot analysis of the total protein expression levels of MT1-MMP in MDA-MB-436 cells cultured for 24 hours with the indicated agents. Data are shown in arbitrary units (AU) normalized to PBS as the mean ± SD of three independent experiments. # P<0.01 as compared to PBS.
(TIF)

Figure S4 **HGF results in PI3K/Akt pathway phosphorylation and activates PKCζ phosphorylation.** (A). Detection of phosphorylated Akt or of the respective total Akt protein expression or Rac1-GTP and total-Rac1 by western blot analysis. MDA-MB-436 cells were left with PBS or were exposed to HGF/SF (for 10 minutes at 50 ng/ml) after preincubation with the PI3-kinase inhibitors wortmannin (at 100 nM, for 60 min) or LY 294002 (at 10 µM, for 60 min) as indicated. Data are shown in arbitrary units (AU) normalized to PBS as the mean ± SD of three independent experiments. # P<0.01 as compared to PBS. (B). MDA-MB-436 cells were exposed to HGF with or without PI 3-kinase inhibitor LY294002 (30 µM) or Akt inhibitor III (50 µM) for various amounts of time, which resulted in the phosphorylation of PKCζ. Data are shown in arbitrary units (AU) normalized to PBS as the mean ± SD of three independent experiments. * indicates P<0.05, # indicates P<0.01 as compared to PBS.
(TIF)

Figure S5 **Treatment with HGF slightly enhanced the tumor growth of breast cancer xenografts in BALB/c-nu mice.** Tumor volume in the mammary fat pads was monitored in BALB/c-nu mice xenografted with MDA-MB-436 (2×10^6^ cells) breast cancer cells that were uninfected or infected with GFP-shRNA or PKCζ-shRNA. Biweekly intratumoral injection with PBS or 30 µg/kg HGF was performed for 4 consecutive weeks once the xenografts were palpable (around 5 mm in diameter). The number of mice with detectable tumors is indicated.
(TIF)

Figure S6 **HGF enhances CXCR4 expression via PKCζ and promotes the invasion and metastasis of breast cancers in BALB/c-nu mice.** (A–B) Immunoblot of total PKCζ and p-PKCζ (A) or total CXCR4 and p-CXCR4 (B) protein, respectively, in breast tumor xenografts in female nude mice that were inoculated in the mammary fat pads with MDA-MB-436 cells treated as indicated. Data are shown in arbitrary units (AU) normalized to PBS as the mean ± SD of three independent experiments. # indicates P<0.01 as compared to PBS.
(TIF)

Figure S7 **Expression of phosphorylated PKCζ or phosphorylated CXCR4 was reduced in breast cancer cells transduced with PKCζ-shRNA.** (A–B)Breast cancer cells expressing phosphorylated-PKCζ (A) or phosphorylated-CXCR4 (B) were count per field of view and were determined by immunohistochemical staining of tumor lesions from mice bearing breast cancer xenografts transduced with PKCζ-shRNA. # P<0.01 as compared to PBS-treated mice.
(TIF)

Figure S8 **HGF enhances CXCR4 expression via PKCζ and promotes the invasion and metastasis of breast cancers in BALB/c-nu mice.** Representative micrographs of the lungs and livers of each group from two independent experiments.
(TIF)

Table S1 **CXCR4 and phospho-c-Met^+^ counts as related to clinicopathological status in 197 cases of breast cancer patients.** Note: *, grading in 197 cases of invasive ductal carcinoma; #, distant metastasis was identified during postoperative follow-up.
(DOC)

Table S2 **Incidence of tumors from MDA-MB-436 cells inoculated in BALB/c-nu mice.** Notes: *, P<0.05 vs. PBS.
(DOC)

Table S3 **Primers for qRT-PCR.**
(DOC)

## References

[1] Chen J, Yao Y, Gong C, Yu F, Su S CCL18 from tumor-associated macrophages promotes breast cancer metastasis via PITPNM3.. Cancer Cell.

[2] Yu F, Yao H, Zhu P, Zhang X, Pan Q (2007). let-7 regulates self renewal and tumorigenicity of breast cancer cells.. Cell.

[3] Balkwill F (2004). The significance of cancer cell expression of the chemokine receptor CXCR4.. Semin Cancer Biol.

[4] Balkwill F (2004). Cancer and the chemokine network.. Nat Rev Cancer.

[5] Meier R, Muhlethaler-Mottet A, Flahaut M, Coulon A, Fusco C (2007). The chemokine receptor CXCR4 strongly promotes neuroblastoma primary tumour and metastatic growth, but not invasion.. PLoS One.

[6] Zlotnik A (2004). Chemokines in neoplastic progression.. Semin Cancer Biol.

[7] Muller A, Homey B, Soto H, Ge N, Catron D (2001). Involvement of chemokine receptors in breast cancer metastasis.. Nature.

[8] Gerlach LO, Skerlj RT, Bridger GJ, Schwartz TW (2001). Molecular interactions of cyclam and bicyclam non-peptide antagonists with the CXCR4 chemokine receptor.. J Biol Chem.

[9] Signoret N, Oldridge J, Pelchen-Matthews A, Klasse PJ, Tran T (1997). Phorbol esters and SDF-1 induce rapid endocytosis and down modulation of the chemokine receptor CXCR4.. J Cell Biol.

[10] Busillo JM, Armando S, Sengupta R, Meucci O, Bouvier M Site-specific phosphorylation of CXCR4 is dynamically regulated by multiple kinases and results in differential modulation of CXCR4 signaling.. J Biol Chem.

[11] Goichberg P, Kalinkovich A, Borodovsky N, Tesio M, Petit I (2006). cAMP-induced PKCzeta activation increases functional CXCR4 expression on human CD34+ hematopoietic progenitors.. Blood.

[12] Wang J, Guan E, Roderiquez G, Calvert V, Alvarez R (2001). Role of tyrosine phosphorylation in ligand-independent sequestration of CXCR4 in human primary monocytes-macrophages.. J Biol Chem.

[13] Tyan SW, Kuo WH, Huang CK, Pan CC, Shew JY Breast cancer cells induce cancer-associated fibroblasts to secrete hepatocyte growth factor to enhance breast tumorigenesis.. PLoS One.

[14] Comoglio PM, Boccaccio C (2001). Scatter factors and invasive growth.. Semin Cancer Biol.

[15] Eckerich C, Zapf S, Fillbrandt R, Loges S, Westphal M (2007). Hypoxia can induce c-Met expression in glioma cells and enhance SF/HGF-induced cell migration.. Int J Cancer.

[16] Ma PC, Tretiakova MS, Nallasura V, Jagadeeswaran R, Husain AN (2007). Downstream signalling and specific inhibition of c-MET/HGF pathway in small cell lung cancer: implications for tumour invasion.. Br J Cancer.

[17] Maroni P, Bendinelli P, Matteucci E, Desiderio MA (2007). HGF induces CXCR4 and CXCL12-mediated tumor invasion through Ets1 and NF-kappaB.. Carcinogenesis.

[18] Matteucci E, Bendinelli P, Desiderio MA (2009). Nuclear localization of active HGF receptor Met in aggressive MDA-MB231 breast carcinoma cells.. Carcinogenesis.

[19] Nagy J, Curry GW, Hillan KJ, McKay IC, Mallon E (1996). Hepatocyte growth factor/scatter factor expression and c-met in primary breast cancer.. Surg Oncol.

[20] Ridolfi E, Matteucci E, Maroni P, Desiderio MA (2008). Inhibitory effect of HGF on invasiveness of aggressive MDA-MB231 breast carcinoma cells, and role of HDACs.. Br J Cancer.

[21] Esencay M, Newcomb EW, Zagzag D HGF upregulates CXCR4 expression in gliomas via NF-kappaB: implications for glioma cell migration.. J Neurooncol.

[22] Tu H, Zhou Z, Liang Q, Li Z, Li D (2009). CXCR4 and SDF-1 production are stimulated by hepatocyte growth factor and promote glioma cell invasion.. Onkologie.

[23] Chianale F, Rainero E, Cianflone C, Bettio V, Pighini A Diacylglycerol kinase alpha mediates HGF-induced Rac activation and membrane ruffling by regulating atypical PKC and RhoGDI.. Proc Natl Acad Sci U S A.

[24] Liu WS, Heckman CA (1998). The sevenfold way of PKC regulation.. Cell Signal.

[25] Petit I, Goichberg P, Spiegel A, Peled A, Brodie C (2005). Atypical PKC-zeta regulates SDF-1-mediated migration and development of human CD34+ progenitor cells.. J Clin Invest.

[26] Duran A, Rodriguez A, Martin P, Serrano M, Flores JM (2004). Crosstalk between PKCzeta and the IL4/Stat6 pathway during T-cell-mediated hepatitis.. Embo J.

[27] Sun R, Gao P, Chen L, Ma D, Wang J (2005). Protein kinase C zeta is required for epidermal growth factor-induced chemotaxis of human breast cancer cells.. Cancer Res.

[28] Vasavada RC, Wang L, Fujinaka Y, Takane KK, Rosa TC (2007). Protein kinase C-zeta activation markedly enhances beta-cell proliferation: an essential role in growth factor mediated beta-cell mitogenesis.. Diabetes.

[29] Stuart KA, Riordan SM, Lidder S, Crostella L, Williams R (2000). Hepatocyte growth factor/scatter factor-induced intracellular signalling.. Int J Exp Pathol.

[30] Allinen M, Beroukhim R, Cai L, Brennan C, Lahti-Domenici J (2004). Molecular characterization of the tumor microenvironment in breast cancer.. Cancer Cell.

[31] Gschwendt M, Kittstein W, Johannes FJ (1998). Differential effects of suramin on protein kinase C isoenzymes. A novel tool for discriminating protein kinase C activities.. FEBS Lett.

[32] Royal I, Lamarche-Vane N, Lamorte L, Kaibuchi K, Park M (2000). Activation of cdc42, rac, PAK, and rho-kinase in response to hepatocyte growth factor differentially regulates epithelial cell colony spreading and dissociation.. Mol Biol Cell.

[33] Tunggal JA, Helfrich I, Schmitz A, Schwarz H, Gunzel D (2005). E-cadherin is essential for in vivo epidermal barrier function by regulating tight junctions.. Embo J.

[34] Gao Y, Dickerson JB, Guo F, Zheng J, Zheng Y (2004). Rational design and characterization of a Rac GTPase-specific small molecule inhibitor.. Proc Natl Acad Sci U S A.

[35] Haribabu B, Richardson RM, Fisher I, Sozzani S, Peiper SC (1997). Regulation of human chemokine receptors CXCR4. Role of phosphorylation in desensitization and internalization.. J Biol Chem.

[36] Cheng ZJ, Zhao J, Sun Y, Hu W, Wu YL (2000). beta-arrestin differentially regulates the chemokine receptor CXCR4-mediated signaling and receptor internalization, and this implicates multiple interaction sites between beta-arrestin and CXCR4.. J Biol Chem.

[37] Woerner BM, Warrington NM, Kung AL, Perry A, Rubin JB (2005). Widespread CXCR4 activation in astrocytomas revealed by phospho-CXCR4-specific antibodies.. Cancer Res.

[38] Beviglia L, Matsumoto K, Lin CS, Ziober BL, Kramer RH (1997). Expression of the c-Met/HGF receptor in human breast carcinoma: correlation with tumor progression.. Int J Cancer.

[39] Jiang WG, Davies G, Martin TA, Parr C, Watkins G (2006). Expression of membrane type-1 matrix metalloproteinase, MT1-MMP in human breast cancer and its impact on invasiveness of breast cancer cells.. Int J Mol Med.

[40] Kim JI, Cordova AC, Hirayama Y, Madri JA, Sumpio BE (2008). Differential effects of shear stress and cyclic strain on Sp1 phosphorylation by protein kinase Czeta modulates membrane type 1-matrix metalloproteinase in endothelial cells.. Endothelium.

[41] Bartolome RA, Ferreiro S, Miquilena-Colina ME, Martinez-Prats L, Soto-Montenegro ML (2009). The chemokine receptor CXCR4 and the metalloproteinase MT1-MMP are mutually required during melanoma metastasis to lungs.. Am J Pathol.

[42] Shirvaikar N, Marquez-Curtis LA, Ratajczak MZ, Janowska-Wieczorek A Hyaluronic acid and thrombin upregulate MT1-MMP through PI3K and Rac-1 signaling and prime the homing-related responses of cord blood hematopoietic stem/progenitor cells.. Stem Cells Dev.

[43] Zhuge Y, Xu J (2001). Rac1 mediates type I collagen-dependent MMP-2 activation. role in cell invasion across collagen barrier.. J Biol Chem.

[44] Bourbon NA, Yun J, Kester M (2000). Ceramide directly activates protein kinase C zeta to regulate a stress-activated protein kinase signaling complex.. J Biol Chem.

[45] Chung CY, Potikyan G, Firtel RA (2001). Control of cell polarity and chemotaxis by Akt/PKB and PI3 kinase through the regulation of PAKa.. Mol Cell.

[46] Xiao GH, Jeffers M, Bellacosa A, Mitsuuchi Y, Vande Woude GF (2001). Anti-apoptotic signaling by hepatocyte growth factor/Met via the phosphatidylinositol 3-kinase/Akt and mitogen-activated protein kinase pathways.. Proc Natl Acad Sci U S A.

[47] Higuchi M, Masuyama N, Fukui Y, Suzuki A, Gotoh Y (2001). Akt mediates Rac/Cdc42-regulated cell motility in growth factor-stimulated cells and in invasive PTEN knockout cells.. Curr Biol.

[48] Kanazawa S, Fujiwara T, Matsuzaki S, Shingaki K, Taniguchi M bFGF regulates PI3-kinase-Rac1-JNK pathway and promotes fibroblast migration in wound healing.. PLoS One.

[49] Manning G, Plowman GD, Hunter T, Sudarsanam S (2002). Evolution of protein kinase signaling from yeast to man.. Trends Biochem Sci.

[50] Schnelzer A, Prechtel D, Knaus U, Dehne K, Gerhard M (2000). Rac1 in human breast cancer: overexpression, mutation analysis, and characterization of a new isoform, Rac1b.. Oncogene.

[51] Sulpice E, Ding S, Muscatelli-Groux B, Berge M, Han ZC (2009). Cross-talk between the VEGF-A and HGF signalling pathways in endothelial cells.. Biol Cell.

[52] Wysoczynski M, Reca R, Ratajczak J, Kucia M, Shirvaikar N (2005). Incorporation of CXCR4 into membrane lipid rafts primes homing-related responses of hematopoietic stem/progenitor cells to an SDF-1 gradient.. Blood.

[53] Noda Y, Takeya R, Ohno S, Naito S, Ito T (2001). Human homologues of the Caenorhabditis elegans cell polarity protein PAR6 as an adaptor that links the small GTPases Rac and Cdc42 to atypical protein kinase C.. Genes Cells.

[54] Ramos-Nino ME, Blumen SR, Sabo-Attwood T, Pass H, Carbone M (2008). HGF mediates cell proliferation of human mesothelioma cells through a PI3K/MEK5/Fra-1 pathway.. Am J Respir Cell Mol Biol.

[55] Webb CP, Taylor GA, Jeffers M, Fiscella M, Oskarsson M (1998). Evidence for a role of Met-HGF/SF during Ras-mediated tumorigenesis/metastasis.. Oncogene.

[56] McBain VA, Forrester JV, McCaig CD (2003). HGF, MAPK, and a small physiological electric field interact during corneal epithelial cell migration.. Invest Ophthalmol Vis Sci.

[57] Giagulli C, Scarpini E, Ottoboni L, Narumiya S, Butcher EC (2004). RhoA and zeta PKC control distinct modalities of LFA-1 activation by chemokines: critical role of LFA-1 affinity triggering in lymphocyte in vivo homing.. Immunity.

[58] Yamazaki D, Kurisu S, Takenawa T (2009). Involvement of Rac and Rho signaling in cancer cell motility in 3D substrates.. Oncogene.

[59] Gong C, Yao H, Liu Q, Chen J, Shi J Markers of tumor-initiating cells predict chemoresistance in breast cancer.. PLoS One.

