# Supplementary figures and images for: HGF-Induced PKCζ Activation Increases Functional CXCR4 Expression in Human Breast Cancer Cells
Source: PLoS One. 2012 Jan 5;7(1):e29124. doi: 10.1371/journal.pone.0029124 (PMC3252308; doi:10.1371/journal.pone.0029124)

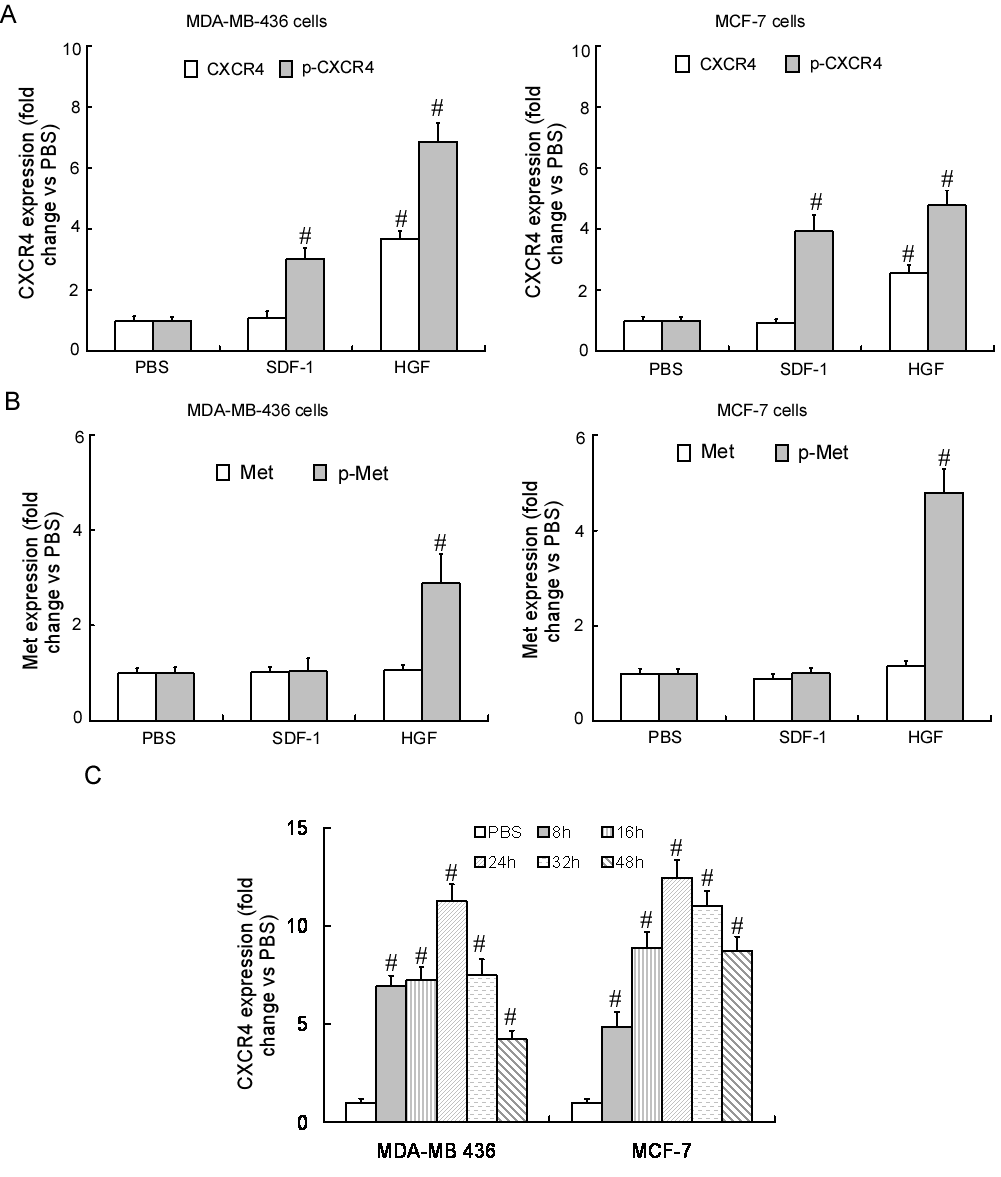

Supplement: Figure S1 — HGF upregulates CXCR4 expression and membrane presentation in human breast cancer cells. (A). Western blotting analysis revealed that the protein levels of CXCR4 and p-CXCR4 treated with HGF were higher than those treated with SDF in MDA-MB- 436 (left panel) and MCF-7 cells (right panel). Data are shown in arbitrary units (AU) normalized to PBS as the mean ± SD of three independent experiments. # P<0.01 as compared to PBS. (B). Western blotting analysis revealed that the protein levels of Met and p-Met treated with HGF were higher than those treated with SDF in MDA-MB- 436 (left panel) and MCF-7 cells (right panel). Data are shown in arbitrary units (AU) normalized to PBS as the mean ± SD of three independent experiments. # P<0.01 as compared to PBS. (C).Time course of relative CXCR4 content in two breast cancer cells following 50 ng/ml HGF stimulation. Data are shown in arbitrary units (AU) normalized to PBS as the mean ± SD of three independent experiments. # P<0.01 as compared to PBS. (TIF) [file pone.0029124.s001.tif]

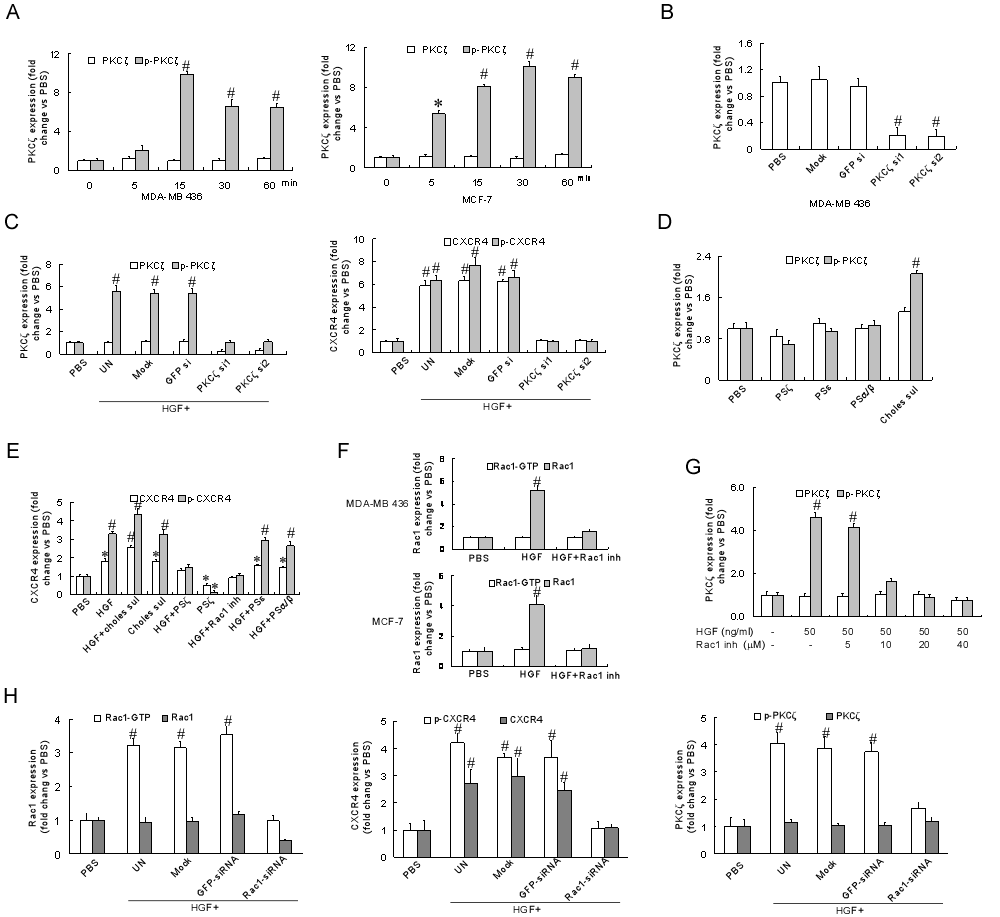

Supplement: Figure S2 — HGF-induced increase in CXCR4 expression depends on PKCζ activity. (A). Time course of relative p-PKCζ levels as determined by immunoblot in MDA-MB-436 (left panel) and MCF-7 cells(right panel) following stimulation with 50 ng/ml HGF. Data are shown in arbitrary units (AU) normalized to PBS as the mean ± SD of three independent experiments. # P<0.01, *P<0.05 as compared to PBS. (B).100 µM PKCζ-siRNA1 (si1) or PKCζ-siRNA2 (si2) transient transfections and siRNA-mediated PKCζ protein silencing in MDA-MB-436 cells. Data are shown in arbitrary units (AU) normalized to PBS as the mean ± SD of three independent experiments. # P<0.01 as compared to PBS. (C). Western blot analysis of PKCζ(left panel) and CXCR4(right panel)expression levels in MDA-MB-436 cells cultured for 24 hours with 50 ng/ml HGF or/and 100 µM PKCζ-siRNA1 (si1) or PKCζ-siRNA2 (si2). Data are shown in arbitrary units (AU) normalized to PBS as the mean ± SD of three independent experiments. # P<0.01 as compared to PBS. (D) and (E). Western blot analysis of total CXCR4 expression and p-CXCR4 levels in MDA-MB-436 cells cultured for 24 hours with 50 ng/ml HGF, 2 µM cholesterol sulfate (Choles sul), or PBS. As indicated, 10 µM PS of PKCζ (PSζ), PKCε (PSε), or PKCα/β (PSα/β) or 25 µM NSC23766 was used. Data are shown in arbitrary units (AU) normalized to PBS as the mean ± SD of three independent experiments. # P<0.01*P<0.05 as compared to PBS. (F). Western blot analysis of Rac1-GTP and total-Rac1 in MDA-MB-436 and MCF-7 cells treated with 50 ng/ml HGF with or without 25 µM NSC23766. The experiment was repeated three times with similar results. A representative study is shown. # P<0.01 as compared to PBS. (G). Dose-dependent inhibition of HGF-induced p-PKCζ was achieved using NSC23766 in HGF-treated MDA-MB-436 cells; the cells were assayed by Western blot. Data are shown in arbitrary units (AU) normalized to PBS as the mean ± SD of three independent experiments. # P<0.01 as compared to PBS. (H). Western blot anal [file pone.0029124.s002.tif]

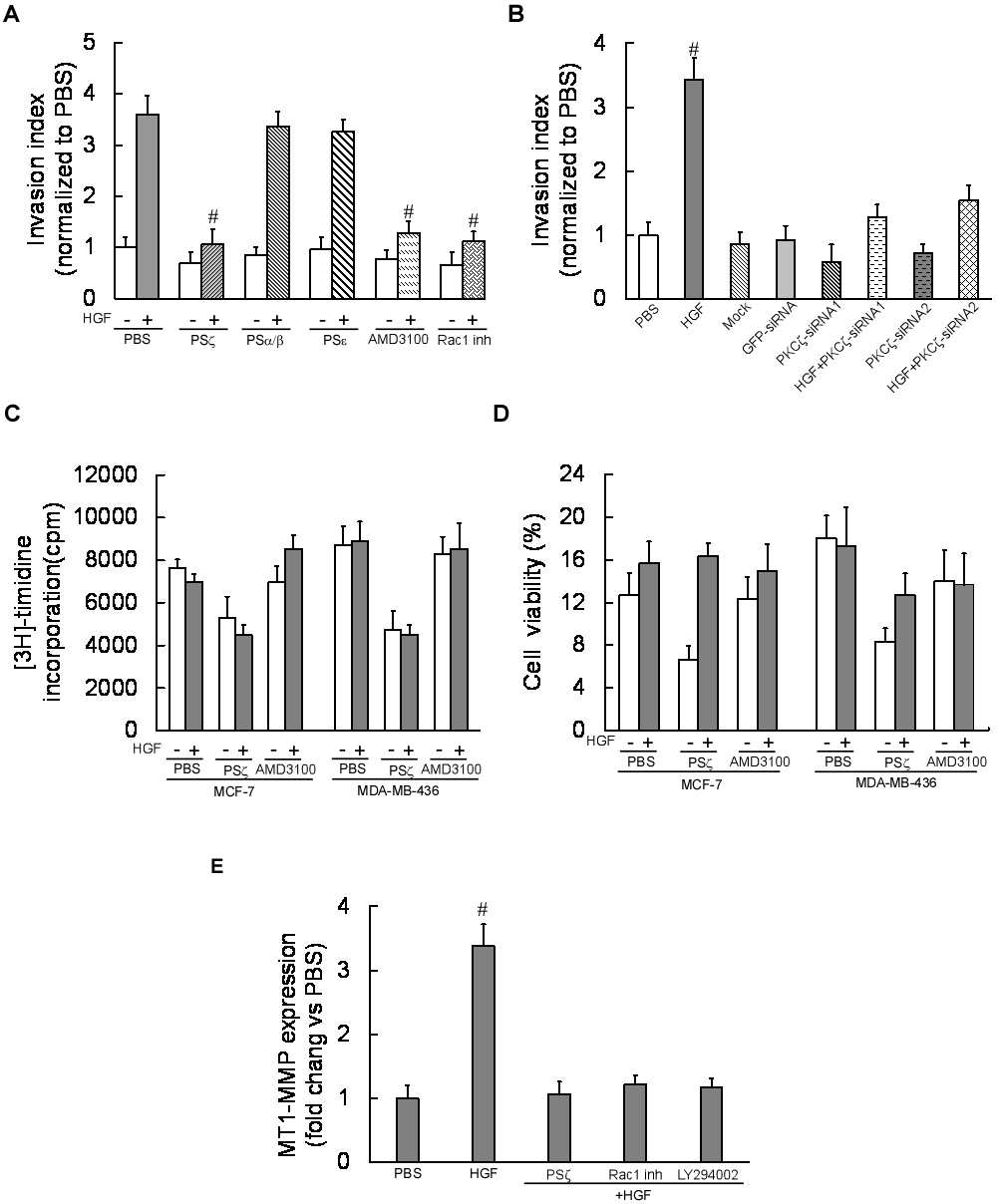

Supplement: Figure S3 — Overexpressed CXCR4 in HGF-stimulated MDA-MB-436 cells is functional. (A–B). MDA-MB-436 cells were treated with indicated agents determined by Boyden chamber assays. Cells were counted in triplicate wells and in three identical experiments. Data are shown in invasion index normalized to PBS as the mean ± SD of three independent experiments. # P<0.01 as compared to PBS. (C). Effect of HGF on breast cancer cell proliferation. MDA-MB 436 and MCF-7 cell lines were cultured for 3 days in serum-free medium with HGF (50 ng/ml) and/or other indicated agents, [3H]-thymidine (1 µCi/well; Amersham Biosciences) was added for an additional 16 hours. Cells were harvested onto fiberglass filters, and radioactivity was detected in a Matrix-96 direct β counter (Packard Instrument Co.) Results shown are representative of three independent experiments. (D). For the MDA-MB 436 and MCF-7 cells were cultured in the presence of HGF and/or other indicated agents for 4 days. Cell viability was determined by MTS assay and reported as a percent of untreated cells. Results shown are representative of three independent experiments. (E). Western blot analysis of the total protein expression levels of MT1-MMP in MDA-MB-436 cells cultured for 24 hours with the indicated agents. Data are shown in arbitrary units (AU) normalized to PBS as the mean ± SD of three independent experiments. # P<0.01 as compared to PBS. (TIF) [file pone.0029124.s003.tif]

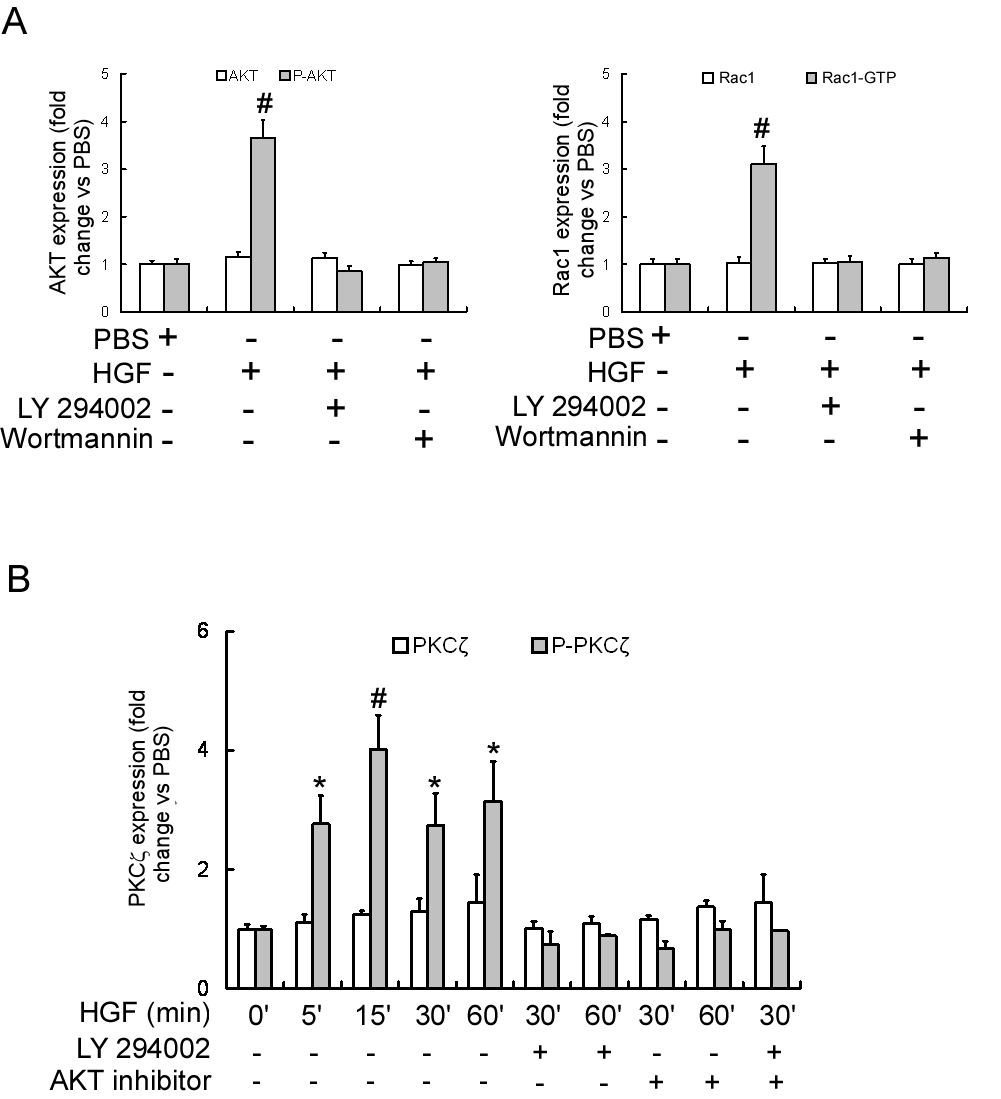

Supplement: Figure S4 — HGF results in PI3K/Akt pathway phosphorylation and activates PKCζ phosphorylation. (A). Detection of phosphorylated Akt or of the respective total Akt protein expression or Rac1-GTP and total-Rac1 by western blot analysis. MDA-MB-436 cells were left with PBS or were exposed to HGF/SF (for 10 minutes at 50 ng/ml) after preincubation with the PI3-kinase inhibitors wortmannin (at 100 nM, for 60 min) or LY 294002 (at 10 µM, for 60 min) as indicated. Data are shown in arbitrary units (AU) normalized to PBS as the mean ± SD of three independent experiments. # P<0.01 as compared to PBS. (B). MDA-MB-436 cells were exposed to HGF with or without PI 3-kinase inhibitor LY294002 (30 µM) or Akt inhibitor III (50 µM) for various amounts of time, which resulted in the phosphorylation of PKCζ. Data are shown in arbitrary units (AU) normalized to PBS as the mean ± SD of three independent experiments. * indicates P<0.05, # indicates P<0.01 as compared to PBS. (TIF) [file pone.0029124.s004.tif]

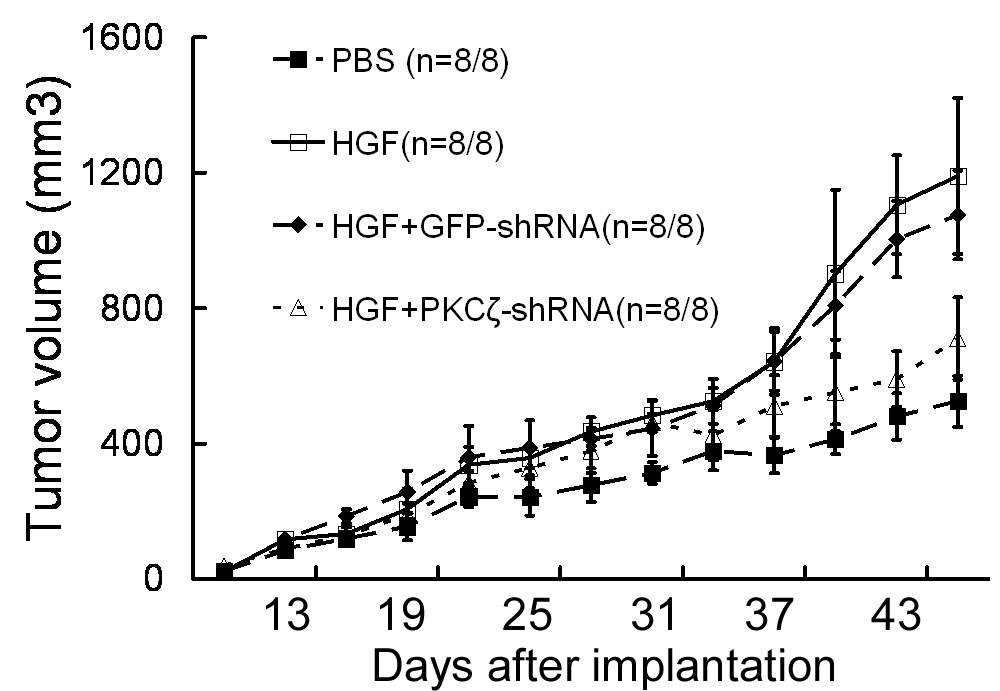

Supplement: Figure S5 — Treatment with HGF slightly enhanced the tumor growth of breast cancer xenografts in BALB/c-nu mice. Tumor volume in the mammary fat pads was monitored in BALB/c-nu mice xenografted with MDA-MB-436 (2×10^6^ cells) breast cancer cells that were uninfected or infected with GFP-shRNA or PKCζ-shRNA. Biweekly intratumoral injection with PBS or 30 µg/kg HGF was performed for 4 consecutive weeks once the xenografts were palpable (around 5 mm in diameter). The number of mice with detectable tumors is indicated. (TIF) [file pone.0029124.s005.tif]

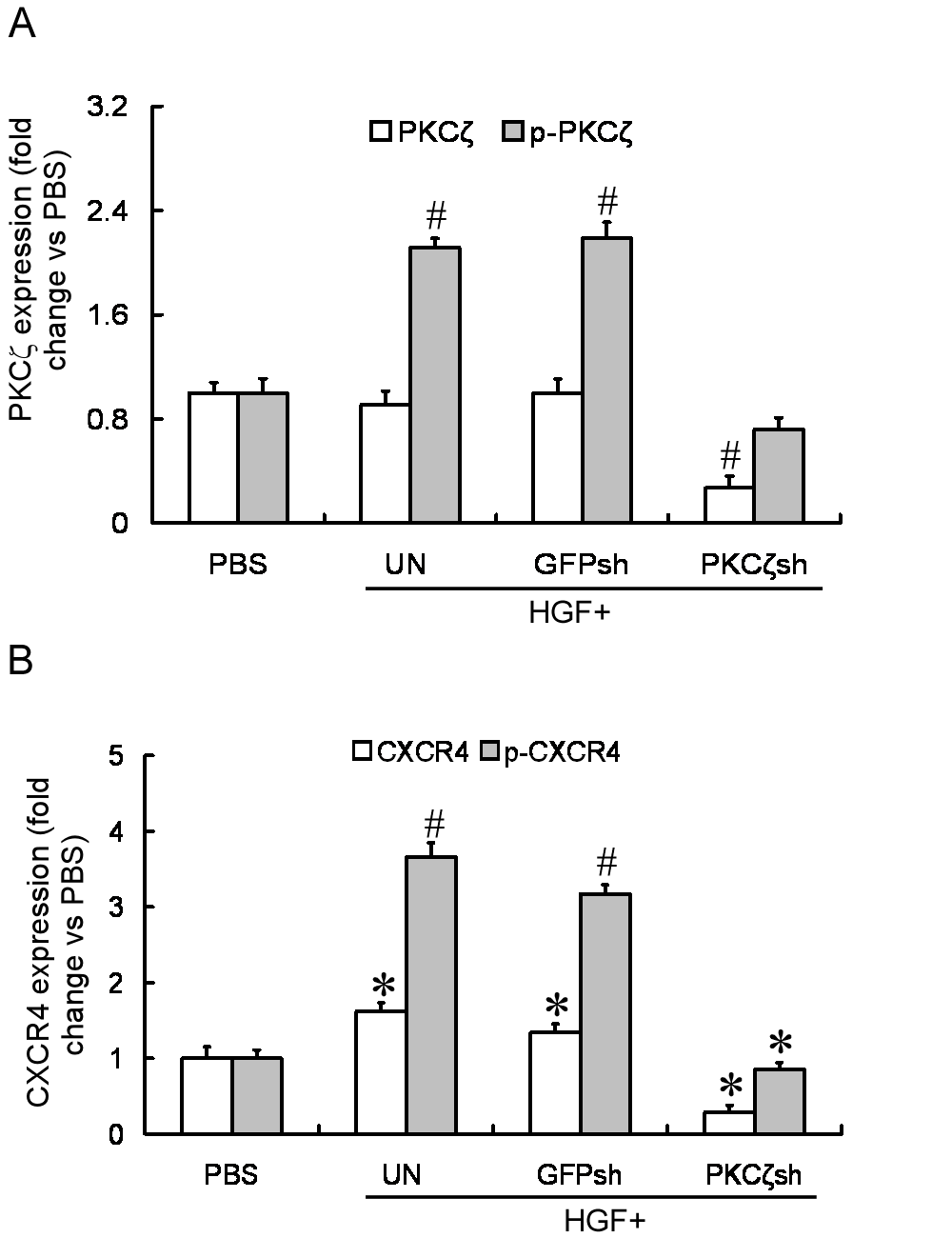

Supplement: Figure S6 — HGF enhances CXCR4 expression via PKCζ and promotes the invasion and metastasis of breast cancers in BALB/c-nu mice. (A–B) Immunoblot of total PKCζ and p-PKCζ (A) or total CXCR4 and p-CXCR4 (B) protein, respectively, in breast tumor xenografts in female nude mice that were inoculated in the mammary fat pads with MDA-MB-436 cells treated as indicated. Data are shown in arbitrary units (AU) normalized to PBS as the mean ± SD of three independent experiments. # indicates P<0.01 as compared to PBS. (TIF) [file pone.0029124.s006.tif]

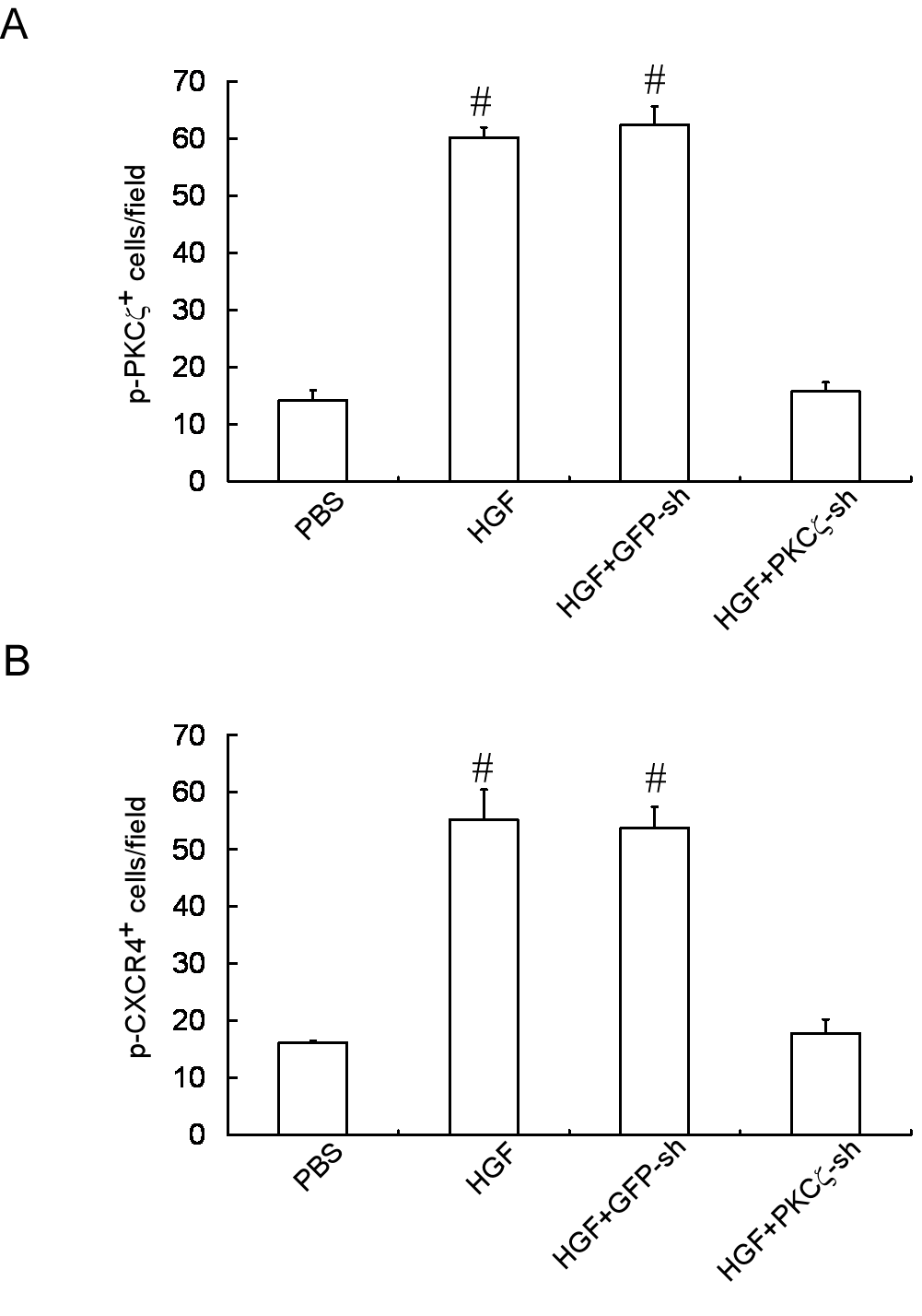

Supplement: Figure S7 — Expression of phosphorylated PKCζ or phosphorylated CXCR4 was reduced in breast cancer cells transduced with PKCζ-shRNA. (A–B)Breast cancer cells expressing phosphorylated-PKCζ (A) or phosphorylated-CXCR4 (B) were count per field of view and were determined by immunohistochemical staining of tumor lesions from mice bearing breast cancer xenografts transduced with PKCζ-shRNA. # P<0.01 as compared to PBS-treated mice. (TIF) [file pone.0029124.s007.tif]

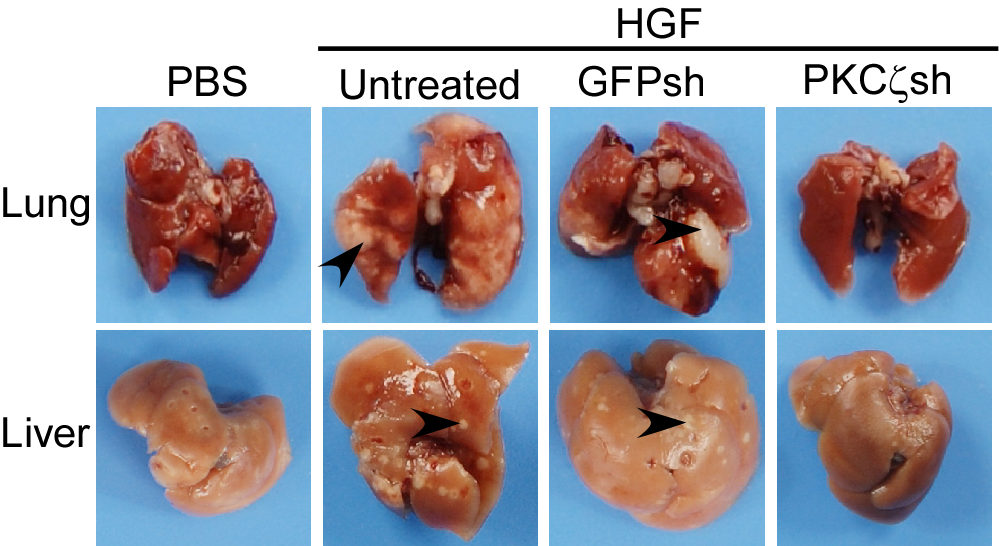

Supplement: Figure S8 — HGF enhances CXCR4 expression via PKCζ and promotes the invasion and metastasis of breast cancers in BALB/c-nu mice. Representative micrographs of the lungs and livers of each group from two independent experiments. (TIF) [file pone.0029124.s008.tif]
