# Supplementary material for: HGF-Induced PKCζ Activation Increases Functional CXCR4 Expression in Human Breast Cancer Cells
Source: PLoS One. 2012 Jan 5;7(1):e29124. doi: 10.1371/journal.pone.0029124 (PMC3252308; doi:10.1371/journal.pone.0029124)
Supplement: Table S1 — CXCR4 and phospho-c-Met+ counts as related to clinicopathological status in 197 cases of breast cancer patients. Note: *, grading in 197 cases of invasive ductal carcinoma; #, distant metastasis was identified during postoperative follow-up. (DOC) [file pone.0029124.s009.doc]

| Variables | CXCR4  <25% ≥25% | | p value | phospho-c-Met  <25% ≥25% | | p value |
| --- | --- | --- | --- | --- | --- | --- |
| Age  ≤45  ＞45  Tumor size (cm)  ≤2  ＞2  Histological grade**﹡**  Ⅰ  Ⅱ  III  Metastasis^#^  (+)  (-)  Lymph node metastasis  0  1-3  ≥4  ER  (+)  (-)  Her2  (+)  (-) | 44  45    49  58  5  62  14  7  92  83  38  1  32  41  12  74 | 53  55  38  52  3  66  67  38  70  4  52  19  72  52  34  77 | 0.959  0.615  <0.001  <0.001  <0.001  0.053  0.006 | 50  33  36  47  7  49  19  2  81    81  33  2  43  39  7  63 | 47  67  44  63  1  79  62  43  71  6  67  18  61  54  39  88 | 0.008  0.755    <0.001  <0.001  <0.001  0.933  0.001 |

**Supplemental Table 1**  **Associations between CXCR4 and phospho-c-Met^+^ counts with clinicopathological status in 197 cases of breast cancer patients**

Note:***,** grading in 197 cases of invasive ductal carcinoma. ^#^, distant metastasis

identified during post-operative follow-up.
