# Supplementary material for: HGF-Induced PKCζ Activation Increases Functional CXCR4 Expression in Human Breast Cancer Cells
Source: PLoS One. 2012 Jan 5;7(1):e29124. doi: 10.1371/journal.pone.0029124 (PMC3252308; doi:10.1371/journal.pone.0029124)
Supplement: Table S2 — Incidence of tumors from MDA-MB-436 cells inoculated in BALB/c-nu mice. Notes: *, P<0.05 vs. PBS. (DOC) [file pone.0029124.s010.doc]

| *Group* | *Tumors* | *Lung metastasis* | **Liver metastasis** |
| --- | --- | --- | --- |
| PBS  HGF  HGF+GFP-shRNA  HGF+PKCζ-shRNA | 8/8  8/8  8/8  8/8 | 1/8  5/8^＊^  4/8  1/8 | 1/8  3/8^＊^  4/8  1/8 |

**Supplemental Table 2 Incidence of tumors from MDA-MB 436 cells inoculated in BALB/c-nu mice^#^**

Notes: #, Number of Cells Inoculated:2×10^6^

^＊^, P<0.05 vs. PBS.
