# Supplementary material for: HGF-Induced PKCζ Activation Increases Functional CXCR4 Expression in Human Breast Cancer Cells
Source: PLoS One. 2012 Jan 5;7(1):e29124. doi: 10.1371/journal.pone.0029124 (PMC3252308; doi:10.1371/journal.pone.0029124)
Supplement: Table S3 — Primers for qRT-PCR. (DOC) [file pone.0029124.s011.doc]

| *Gene* | **Primer (5′-3′)** |
| --- | --- |
| Human CXCR4  Human GADPH  Human HPRT  Mouse 18S rRNA  MT1-MMP | Forward, 5'- TCTAGGCAG-GACCTGT -3'  Reverse, 5′- CACTTTGGGCTTTGGTT -3′  Forward, 5′- TTCACCACCATGGAGAAGGC-3′  Reverse, 5′- GGCATGGACTGTGGTCATGA-3′  Forward, 5′- TTCCTTGGTCAGGCAGTATAATCC-3′  Reverse, 5′- AGTCTGGCTTATATCCAACACTTCG-3′  Forward, 5′- CGGCTACCACATCCAAGGAA-3′  Reverse, 5′- GCTGGAATTACCGCGGCT-3′  Forward, 5′-GCTTGCAAGTAACAGGCAAA-3′  Reverse, 5′-AAATTCTCCGTGTCCATCCA-3′ |

**Supplemental Table 3 Primers for qRT-PCR**
